# The Role of Iron Homeostasis Imbalance in T2DM‐Associated Cognitive Dysfunction: A Prospective Cohort Study Utilizing Quantitative Susceptibility Mapping

**DOI:** 10.1002/hbm.70263

**Published:** 2025-06-18

**Authors:** Zhenyu Cheng, Linfeng Yang, Meng Li, Qihao Zhang, Jing Li, Nan Zhang, Yena Che, Yiwen Chen, Pengcheng Liang, Yuanyuan Wang, Na Wang, Xinyue Zhang, Changhu Liang, Lingfei Guo

**Affiliations:** ^1^ School of Medical Imaging Binzhou Medical University Yantai Shandong China; ^2^ Key Laboratory of Endocrine Glucose & Lipids Metabolism and Brain Aging, Ministry of Education, Department of Radiology Shandong Provincial Hospital Affiliated to Shandong First Medical University Jinan Shandong China; ^3^ Department of Radiology Jinan Maternity and Child Care Hospital Affiliated to Shandong First Medical University Jinan Shandong China; ^4^ Department of Psychiatry and Psychotherapy Jena University Hospital Jena Germany; ^5^ Department of Radiology Weill Cornell Medical College New York USA; ^6^ Department of Radiology Beijing Tsinghua Changgung Hospital Beijing China; ^7^ Department of Clinical Laboratory Shandong Provincial Hospital Affiliated to Shandong First Medical University Jinan Shandong China

**Keywords:** cognitive dysfunction, iron deposition, quantitative susceptibility mapping, subcortical nuclei, type 2 diabetes

## Abstract

Type 2 diabetes mellitus (T2DM) is a chronic metabolic disorder that significantly impacts cognitive health. Although the vascular complications of T2DM have been extensively studied, research on brain iron deposition in T2DM remains scarce, and few studies have directly linked iron accumulation in cognition‐related subcortical nuclei to cognitive dysfunction. This study aims to evaluate brain iron deposition using quantitative susceptibility mapping (QSM) and identify key subcortical nuclei associated with T2DM‐related cognitive decline. A total of 224 participants were recruited, including 112 T2DM patients and 112 healthy controls. QSM was used to assess iron deposition in subcortical nuclei. Structural equation modeling was employed to construct interaction models between metabolic changes, susceptibility values, and cognitive function. Additionally, polynomial regression analysis was performed to evaluate the association between glycemic variability and the QSM values of subcortical nuclei. Our findings confirmed that T2DM patients exhibited pronounced iron deposition in the caudate and putamen compared to healthy controls. Correlation analyses showed that higher QSM values in the anterior putamen, posterior putamen, and posterior caudate were associated with slower processing speed (SDMT), reduced memory performance (AVLT) and poorer executive function (TMT, SCWT), indicating that greater iron accumulation in these nuclei is associated with poorer cognitive performance. In our SEM, metabolic dysregulation was significantly associated with higher subcortical susceptibility (β = 0.224, *p* = 0.010). The model further demonstrated that susceptibility values partially mediated the effect of metabolic factors on cognition (indirect effect *β* = −0.056, *p* = 0.018) and that the overall impact of metabolic dysregulation on cognition remained significant (*β* = −0.142, *p* = 0.037). Polynomial regression found that HbA1c was the strongest predictor of anterior putamen susceptibility, and a similar pattern was observed in the posterior caudate. The study demonstrates that the role of brain iron deposition in T2DM‐related cognitive dysfunction. These findings reveal an important underlying mechanism of T2DM‐induced cognitive impairment and provide evidence for early intervention strategies to mitigate cognitive decline in T2DM patients.

## Graphical abstract

Brain iron deposition in the caudate and putamen is elevated in Type 2 diabetes mellitus (T2DM) and mediates the link between blood glucose fluctuations and cognitive decline. These findings highlight iron homeostasis disruption as a key mechanism in T2DM‐related cognitive impairment, suggesting potential targets for early intervention.

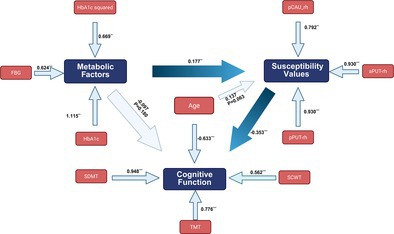

## 1 Introduction

Type 2 diabetes mellitus (T2DM) is a chronic metabolic disorder characterized by insulin resistance and progressive β‐cell dysfunction. The prevalence of T2DM is rapidly increasing, with global cases projected to rise from 463 million in 2019 to 700 million by 2045 (Magliano et al. 2021; NCD Risk Factor Collaboration (NCD‐RisC) 2016). For a long time, it has been widely recognized that in T2DM patients, peripheral insulin resistance and compensatory hyperinsulinemia can lead to a series of complications, such as peripheral neuropathy, nephropathy, atherosclerosis, and retinopathy (Forbes and Cooper 2013; Zhu et al. 2025; Li et al. 2025). However, the impact of diabetes on cognitive function has received relatively little attention.

Cognitive dysfunction is a common complication and comorbidity of both T2DM and type 1 diabetes mellitus (T1DM) (Pignalosa et al. 2021). Studies have indicated that, compared with nondiabetic individuals, T2DM patients have a 30% to 65% increased risk of developing Alzheimer's disease, and the incidence of mild cognitive impairment and dementia nearly doubles, making T2DM a major risk factor for dementia development (Burillo et al. 2021). Furthermore, both prediabetes and diabetes are independently associated with accelerated cognitive decline, particularly in aging populations (Willmann et al. 2020). Current research has focused primarily on the effects of blood glucose on cerebrovascular health, whereas neuronal damage in the brain has often been overlooked. In fact, brain insulin resistance not only disrupts the function of insulin in the nervous system but also may lead to overphosphorylation of tau protein and excessive amyloid deposition, resulting in neuronal damage (Leboucher et al. 2019; Verma et al. 2021). This pathological state is referred to as T2DM‐associated cognitive dysfunction (TDACD), which has become a significant factor affecting the quality of life and life expectancy of T2DM patients. The features of TDACD include cognitive decline, slowed executive function, and impaired information processing speed (Pignalosa et al. 2021).

In recent years, numerous studies have revealed significant structural and functional abnormalities in the brains of T2DM patients, including changes in gray–white matter distribution; abnormal cerebral blood flow perfusion; and structural and functional changes in the striatum and brain regions related to motor control pathways (Li et al. 2020). Among these changes, abnormal iron deposition in the brain has become one of the most significant and crucial alterations. Iron is a key element in many vital physiological processes within the central nervous system (CNS), including oxygen transport, myelination, and neurotransmitter synthesis (Li et al. 2021). However, excessive iron accumulation can be toxic to the body. When iron overload occurs, a cytotoxic positive feedback loop driven by oxidative stress and iron release exacerbates cellular and tissue damage and further amplifies brain tissue pathology through microglial activation (Dusek et al. 2022). Disruption of brain iron metabolism not only directly damages neurons in critical brain regions, destroying axons and synaptic structures but also interferes with communication between neurons, reducing the efficiency of neural networks and thereby impairing motor and cognitive functions (Thirupathi and Chang 2019).

Previous pathological studies have shown that iron metabolic imbalance in the brain occurs early in CNS injury, often preceding significant structural changes in the brain (Mohan et al. 2024; Prakash et al. 2017). Neuroimaging research has further confirmed that changes in brain iron content are more sensitive than structural alterations in detecting brain damage. Traditional quantitative MRI indices of susceptibility values, such as R2* based on relaxometry measurements, estimate the brain iron content by summing the relaxation effects due to spin–spin interactions (R2) and local susceptibility effects (R2′) (Langkammer et al. 2010). However, these methods can be influenced by factors such as background magnetic field inhomogeneities, which may affect the accuracy of iron content estimation (Ngo et al. 2019). Quantitative susceptibility mapping (QSM) offers a direct measure of tissue magnetic susceptibility that is largely driven by non‐heme iron deposition in the brain (Uchida et al. 2022; Uchida, Kan, Furukawa, et al. 2023). However, QSM signals can also be influenced by diamagnetic components such as myelin and protein aggregates (Deh et al. 2018; Ahmed et al. 2023). To reduce these confounds, we applied the MEDI algorithm with an automatic uniform cerebrospinal fluid (CSF) zero reference (MEDI+0) and limited our analyses to iron‐rich deep gray matter nuclei, where paramagnetic iron effects dominate. This approach not only reduces diamagnetic confounds but also exploits the enhanced contrast QSM provides in deep gray regions, enabling precise detection of iron‐related changes in nuclei implicated in TDACD (Barbosa et al. 2015). For example, a prospective study using a strategically acquired gradient echo (STAGE) sequence quantitatively evaluated both iron deposition and volume changes in deep gray nuclei in T2DM patients compared to healthy controls (HCs). This study revealed that magnetic susceptibility values in high‐iron regions of several gray nuclei were increased by 5.1%–14.8% in T2DM, while whole‐structure volumes in most of these nuclei were decreased. Moreover, the regional magnetic susceptibility values (MSVRII) showed a significant correlation with cognitive scores (Hu et al. 2023).

In this study, we aim to use the QSM to precisely measure changes in the susceptibility values of subcortical nuclei in T2DM patients and further track how changes in blood glucose control during the progression of diabetes influence brain iron deposition. Additionally, we will identify specific brain nuclei associated with TDACD damage. Our primary hypothesis is that the progression of diabetes is closely associated with brain iron deposition and that blood glucose fluctuations affect cognitive function by iron homeostasis.

## 2 Method

### 2.1 Participants

This study was approved by the Ethics Committee of Shandong Provincial Hospital, affiliated with Shandong First Medical University, and written informed consent was obtained from all participants. All study procedures were conducted in accordance with the principles of the Declaration of Helsinki and its subsequent amendments. All participants were selected from a large prospective cohort registered under ISRCTN20008650, which was established and is maintained by our research team. The diagnosis of T2DM was confirmed on the basis of a self‐reported physician diagnosis, fasting plasma glucose (FPG) levels ≥ 7.0 mmol/L (126 mg/dL), or glycated hemoglobin (HbA1c) ≥ 6.5%. For individuals with borderline FPG levels (≥ 6.1 mmol/L), a second fasting test was performed after 24 h, and those with persistently elevated glucose levels were diagnosed with T2DM.

**FIGURE 1:**
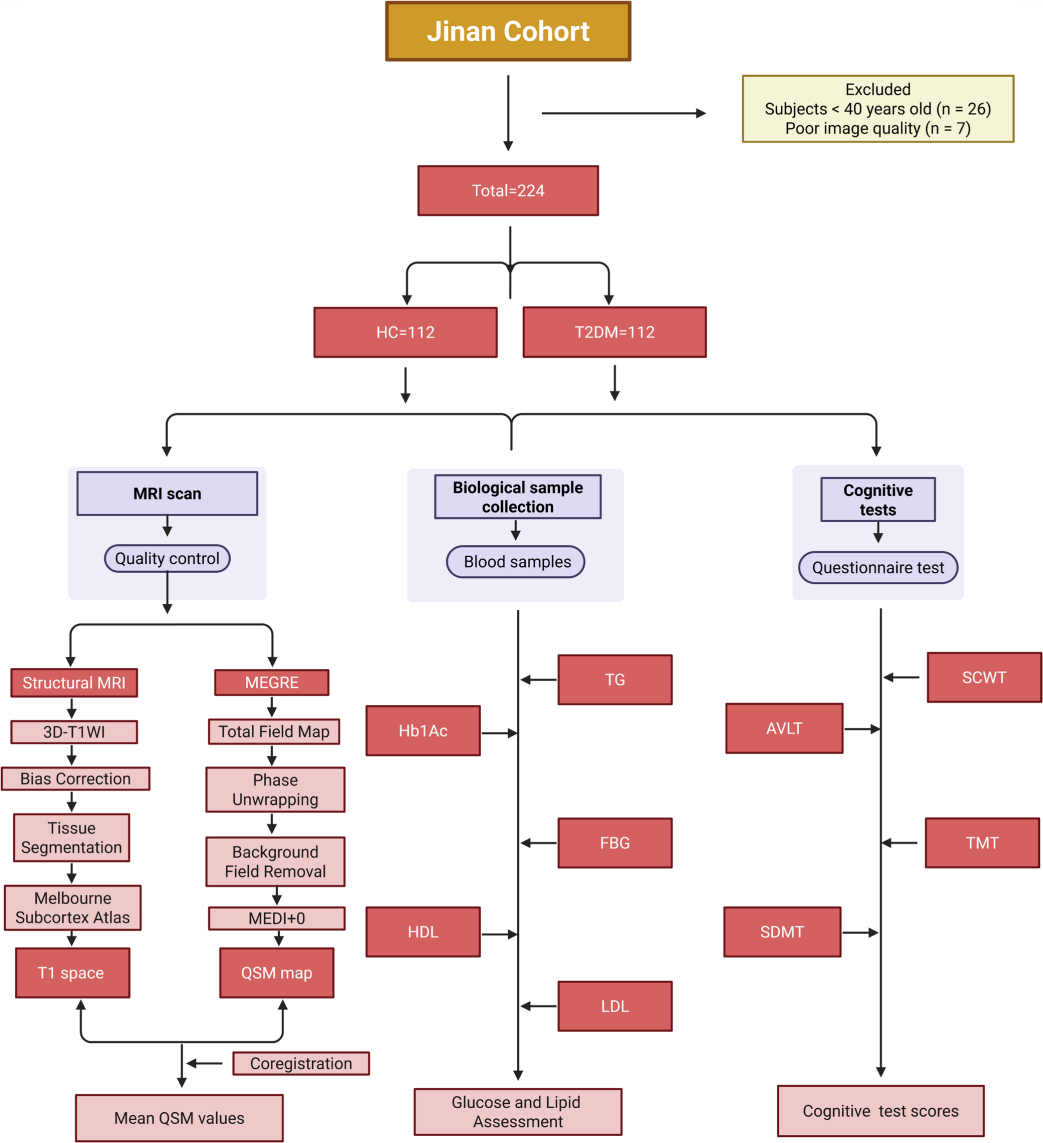
Patient selection and study design. Flow chart of the cohort patient selection process and inclusion and exclusion criteria. AVLT, auditory verbal learning test; FBG, fasting blood glucose; HbA1c, glycated hemoglobin; HDL, high‐density lipoprotein cholesterol; LDL, low‐density lipoprotein cholesterol; MEDI+0, morphology‐enabled dipole Inversion with an automatic uniform cerebrospinal fluid zero reference algorithm; MEDI, morphology‐enabled dipole Inversion; SCWT, Stroop Color and Word Test; SDMT, symbol‐digit modalities test; TG, triglycerides; TMT, trail making test.

A total of 112 T2DM patients and 112 age‐matched HCs were selected from the cohort (Figure 1). The inclusion criteria were as follows: (1) Participants aged 40 years or older. (2) Participants could tolerate MRI scanning without complications. (3) Participants with no history of conditions known to interfere with brain iron metabolism or cause atrophy in subcortical regions, such as brain tumors, multiple sclerosis (MS), Parkinson's disease (PD) or vascular dementia. (4) Participants who had not used psychotropic medications recently to avoid potential effects on subsequent cognitive evaluations. The exclusion criteria were as follows: (1) Poor‐quality MR images: Participants were excluded if image quality hindered accurate postprocessing. (2) Incomplete or significantly biased clinical or imaging data: Participants with incomplete or unreliable data were excluded. (3) Recent diabetes‐related emergencies: Participants who had experienced diabetic ketoacidosis or hyperosmolar coma were excluded. (4) Irregular medication use or failure to report relevant medical history.

We also recruited 112 HCs who met strict exclusion criteria. All HCs completed a structured medical history and lifestyle questionnaire, underwent review of their medical records, and had fasting plasma glucose and HbA1c measured to confirm normal metabolic status. In addition, each HC received a brief neurological examination by a trained clinician to rule out any neurological conditions. MRI images were acquired using standardized protocols, and image quality was independently verified by two radiologists. Clinical and cognitive data were collected and cross‐checked by two independent researchers (Figure 1).

### 2.2 MRI Data Acquisition and Imaging Parameters

MRI data from all participants were acquired using a 3.0‐T MR system (Siemens Healthcare, Erlangen, Germany) equipped with a 32‐channel head coil for signal reception. Three‐dimensional structural images with T1‐weighting (3D T1WI) were acquired using the magnetization‐prepared rapid gradient echo (MPRAGE) sequence with the following parameters: repetition time (TR) = 2300 ms; echo time (TE) = 2.3 ms; flip angle = 9°; field of view (FOV) = 240 × 240 mm^2^; matrix size = 256 × 256; and voxel size = 1 × 1 × 1 mm^3^. Three‐dimensional multiecho gradient‐echo (3D ME‐GRE) images were also acquired to cover the whole brain. The imaging parameters included TR = 50 ms; initial TE = 6.8 ms; echo spacing = 4.1 ms; number of echoes = 10; flip angle = 15°; and voxel size = 1 × 1 × 2 mm^3^.

In addition, conventional MRI sequences were acquired to identify potential brain abnormalities: T2‐weighted imaging (T2WI), T2‐weighted fluid‐attenuated inversion recovery (T2W FLAIR), diffusion‐weighted imaging (DWI), susceptibility‐weighted imaging (SWI).

### 2.3 Imaging Processing

**FIGURE 2:**
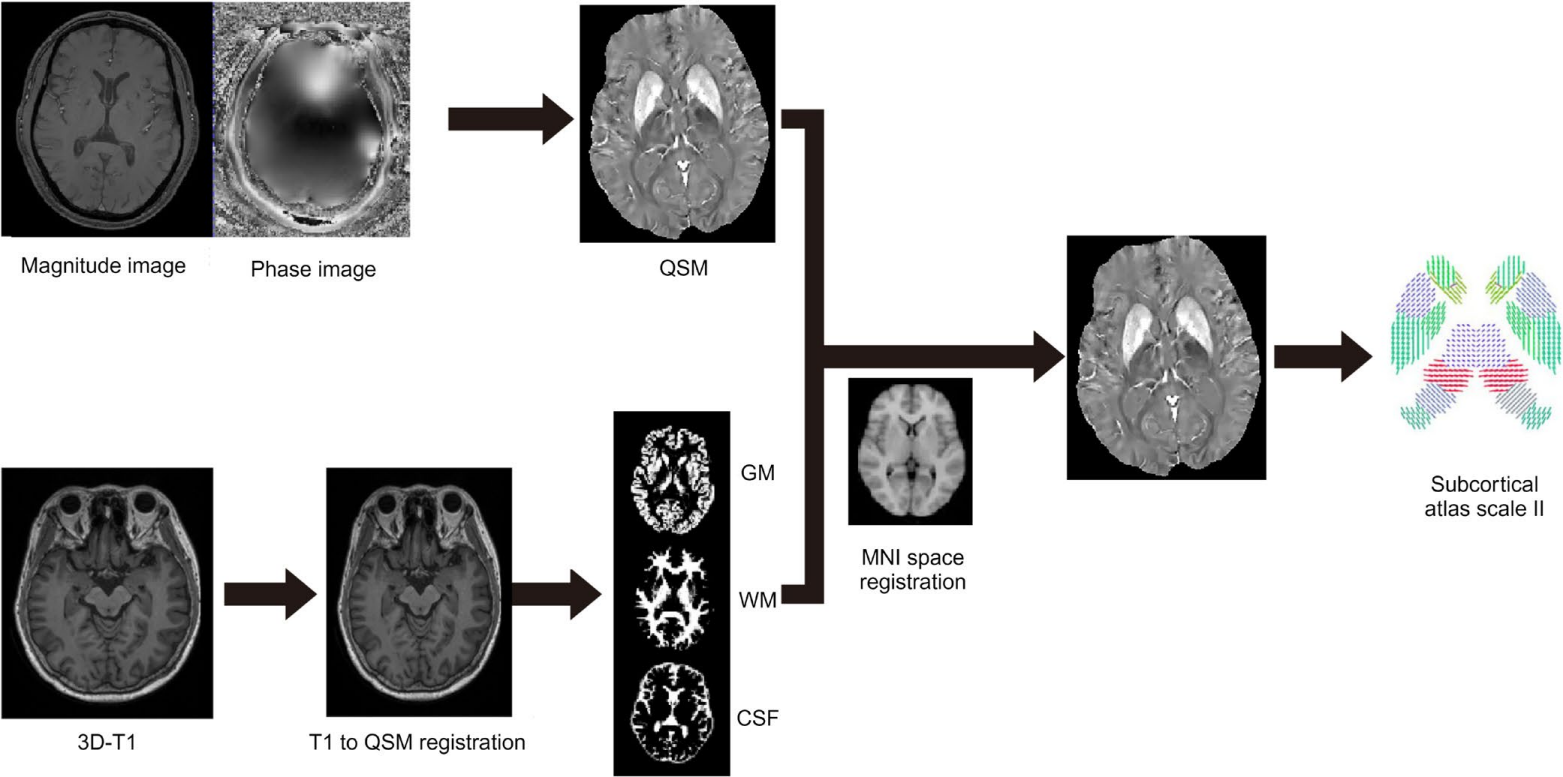
Flowchart of QSM image spatial registration, normalization, and ROI segmentation. By registering QSM images to T1‐weighted images, spatial consistency across different imaging modalities is achieved, enabling precise localization of subcortical regions in the QSM images. Subsequently, using the subcortex atlas derived from the redefinition of functional connectivity gradients and regional boundaries, QSM values for 32 subcortical regions are calculated. 3D‐T1, 3D T1‐weighted imaging; GM, gray matter; MNI, Montreal Neurological Institute; QSM, quantitative susceptibility mapping; WM, white matter.

QSM reconstructions were performed using the morphology‐enabled dipole inversion (MEDI) toolbox (http://pre.weill.cornell.edu/mri/pages/qsm.html) on the MATLAB R2019a (MathWorks, Natick, MA, USA) platform. After complex multiecho gradient‐echo (ME‐GRE) images were obtained, nonlinear fitting was applied to estimate the total magnetic field maps (Liu et al. 2013). Frequency aliasing in the field maps was addressed using a magnitude‐guided spatial unwrapping algorithm (Cusack and Papadakis 2002), and the local tissue field was calculated by removing the background field with the projection onto dipole fields (PDF) method (Liu et al. 2011). The resulting local field was inverted to generate QSM maps using the MEDI algorithm with an automatic uniform cerebrospinal fluid (CSF) zero reference (MEDI+0), which incorporated regularization to minimize susceptibility inhomogeneities in ventricular CSF, improving the accuracy and robustness of the reconstructions (Liu et al. 2018). This integrated pipeline ensured that high‐quality QSM maps were generated for further analyses (Figure 2).

For normalization, the individual MPRAGE image was corrected for intensity nonuniformity and used as the T1‐weighted (T1W) reference. Spatial normalization to the standard space (MNI152NLin2009cAsym) was performed using nonlinear registration with ANTs (v2.2.0), and the resulting transformation matrix was applied to the QSM maps to achieve spatial standardization (Avants et al. 2011).

QSM maps were registered to each subject's T1‐weighted image and normalized to MNI space. We then used the Melbourne Subcortex Atlas to segment 16 subcortical nuclei in each hemisphere (32 ROIs total), including the ventral posterolateral thalamus (THA_VP), dorsoanterior thalamus (THA_DA), nucleus accumbens shell (NAc_shell), nucleus accumbens core (NAc_core), posterior caudate (pCAU), anterior putamen (aPUT), posterior putamen (pPUT), and other regions (Tian et al. 2020). Average magnetic susceptibility values were extracted from all 32 ROIs for further analysis, ensuring a consistent and comprehensive assessment of iron deposition (Figure 2). All QSM values are reported as Δ*χ* × 10^9^ (ppb), referenced to CSF zero via the MEDI+0 algorithm.

### 2.4 Cognitive Assessment

All participants completed several cognitive assessments administered by neurologists trained in cognitive testing, who were blinded to the participants' disease status and other conditions. The Trail Making Test (TMT) includes two components: TMT‐A, which evaluates cognitive function, and TMT‐B, which assesses executive function. Their total completion time was summed to provide a composite TMT score, reflecting overall task performance. The Auditory Verbal Learning Test (AVLT) evaluates episodic memory, encompassing immediate recall, learning ability, and delayed recall of verbally presented information. The Symbol‐Digit Modalities Test (SDMT), a measure of processing speed and visual attention, requires participants to quickly pair symbols with corresponding digits. The Stroop Color and Word Test (SCWT) assesses executive function and attentional control, particularly the ability to resolve interference when naming the ink color of incongruent color words. All tests were conducted in a controlled and quiet environment to minimize distractions.

### 2.5 Statistical Analysis

All the statistical analyses were performed using R software (version 4.3.1). The statistical power analysis was conducted using G*Power software (version 3.1). The analysis was set with the following parameters: Number of response variables = 32, Number of groups = 2, effect size *f* ^2^(V) = 0.20, significance level *α* = 0.05, and target power level 1 − *β* = 0.95. The results indicated that a minimum of 104 participants per group (total sample size = 208) is required to achieve sufficient statistical power to detect significant differences between groups. Our study included a total of 224 participants, with 112 individuals in each group, ensuring adequate statistical power for the analysis.

The Kruskal–Wallis test was employed to compare differences in subcortical nuclei magnetic susceptibility values between the T2DM and HCs. To control the false discovery rate across the 32 ROI comparisons, *p*‐values were adjusted using the Benjamini–Hochberg FDR procedure. For brain nuclei showing significant differences, Spearman correlation analyses were conducted to assess their relationships with cognitive function and identify relevant cognitive measures. To further explore the relationship between glycemic metabolic status and the magnetic susceptibility values of these brain nuclei, restricted cubic spline (RCS) analyses were performed to detect possible nonlinear associations. Significant subcortical nuclei were combined into a latent variable representing magnetic susceptibility values, while selected cognitive measures were similarly combined into a latent variable representing cognitive function. The squared term of HbA1c was included in the model to account for observed nonlinear effects. Mediation effects were assessed using the bootstrap method with 1000 resamples, ensuring robust inference. Finally, polynomial regression was used to assess potential factors influencing the magnetic susceptibility values of the brain nuclei.

## 3 Results

### 3.1 Comparison of Characteristics Between the T2DM and HC Groups

Table 1 presents the demographic and clinical characteristics of the diabetes patients and HCs. The diabetes group had a significantly greater BMI, HbA1c, FBG, and prevalence of hypertension, highlighting metabolic and cardiovascular risks. The cognitive test scores (SDMT, SCWT, and TMT) were significantly lower in the diabetes group than in the control group, suggesting early cognitive impairment. No significant differences were found in MoCA scores or AVLT performance, indicating that certain cognitive functions may remain unaffected in the early stages. Lifestyle factors such as smoking and alcohol consumption were more prevalent in the diabetes group.

**TABLE 1:** Clinical characteristics of the participant.

Variables	Total (*n* = 224)	HC (*n* = 112)	T2DM (*n* = 112)	Statistic	*p*
Age, *M* (*Q*_1_, *Q*_3_)	60.00 (54.00, 66.00)	60.00 (54.00, 65.00)	61.00 (54.00, 66.00)	*Z* = ‐0.53	0.596
BMI, mean ± SD	25.14 ± 3.17	24.63 ± 3.30	25.66 ± 2.96	*t* = −2.45	**0.015**
Education, *M* (*Q*_1_, *Q*_3_)	15.00 (12.00, 16.00)	15.00 (12.00, 17.00)	12.00 (9.00, 16.00)	*Z* = −3.31	**< 0.001**
FBG, *M* (*Q*_1_, *Q*_3_)	6.00 (5.23, 7.83)	5.36 (4.94, 5.81)	7.72 (6.39, 9.99)	*Z* = −10.61	**< 0.001**
HbA1c, *M* (*Q*_1_, *Q*_3_)	6.20 (5.62, 7.90)	5.70 (5.50, 6.00)	7.90 (6.80, 9.60)	*Z* = −12.02	**< 0.001**
TG, *M* (*Q*_1_, *Q*_3_)	1.30 (0.89, 1.87)	1.08 (0.85, 1.60)	1.56 (1.08, 2.11)	*Z* = −3.69	**< 0.001**
HDL, *M* (*Q*_1_, *Q*_3_)	1.37 (1.12, 1.58)	1.50 (1.27, 1.74)	1.20 (1.00, 1.46)	*Z* = −6.12	**< 0.001**
LDL, *M* (*Q*_1_, *Q*_3_)	3.05 (2.49, 3.63)	3.06 (2.52, 3.61)	3.01 (2.46, 3.67)	*Z* = −0.13	0.894
MoCA, *M* (*Q*_1_, *Q*_3_)	25.00 (22.00, 26.00)	25.00 (22.00, 27.00)	24.00 (22.00, 26.00)	*Z* = −1.02	0.308
SDMT, *M* (*Q*_1_, *Q*_3_)	33.00 (24.00, 45.00)	38.00 (26.00, 47.00)	31.00 (22.00, 42.50)	*Z* = −2.36	**0.018**
SCWT, *M* (*Q*_1_, *Q*_3_)	135.00 (113.75, 155.25)	127.00 (110.00, 148.25)	141.50 (121.75, 169.75)	*Z* = −3.20	**0.001**
TMT, *M* (*Q*_1_, *Q*_3_)	216.00 (156.00, 308.00)	197.00 (140.00, 294.85)	238.50 (168.25, 312.75)	*Z* = −2.42	**0.016**
AVLT, mean ± SD	55.03 ± 13.27	54.24 ± 13.42	55.82 ± 13.13	*t* = −0.89	0.376
Gender, *n* (%)				*χ* ^2^ = 14.00	**< 0.001**
Male	112 (50.00)	42 (37.50)	70 (62.50)		
Female	112 (50.00)	70 (62.50)	42 (37.50)		
Hypertension, *n* (%)				*χ* ^2^ = 16.50	**< 0.001**
No	130 (58.04)	80 (71.43)	50 (44.64)		
Yes	94 (41.96)	32 (28.57)	62 (55.36)		
Smoke, *n* (%)				*χ* ^2^ = 13.71	**< 0.001**
No	168 (75.00)	96 (85.71)	72 (64.29)		
Yes	56 (25.00)	16 (14.29)	40 (35.71)		
Drink, *n* (%)				*χ* ^2^ = 7.24	**0.007**
No	149 (66.52)	84 (75.00)	65 (58.04)		
Yes	75 (33.48)	28 (25.00)	47 (41.96)		

*Note:* For clinical and demographic comparisons, normally distributed continuous variables were compared by Student's t‐test, non‐normal variables by Mann–Whitney *U* test, and categorical variables by Chi‐square test. Normality was assessed with the Shapiro–Wilk test. Bold values indicate statistical significance at *p* < 0.05.

Abbreviations: AVLT, auditory verbal learning test; FBG, fasting blood glucose; HbA1c, glycated hemoglobin; HDL, high‐density lipoprotein cholesterol; LDL, low‐density lipoprotein cholesterol; *M*, median; *Q*_1_, 1st quartile; *Q*_3_, 3rd quartile; SCWT, Stroop Color and Word Test; SD, standard deviation; SDMT, symbol‐digit modalities test; *t*, *t*‐test; TG, triglycerides; TMT, trail making test; *Z*, Mann–Whitney test; χ^2^, chi‐square test.

### 3.2 Screening and Adjustment of Differences in Subcortical Nuclei Magnetic Susceptibility Values

Table 2 summarizes the Kruskal–Wallis comparisons of magnetic susceptibility values across all 32 subcortical ROIs. After FDR correction (across those 32 tests), four regions remained significantly elevated in the T2DM group: posterior caudate (pCAU_rh; *χ* ^2^ = 12.48, p_FDR = 0.016), anterior putamen (aPUT_rh; *χ* ^2^ = 10.41, p_FDR = 0.016), dorsoanterior thalamus (THA_DA_rh; *χ* ^2^ = 8.85, p_FDR = 0.032) and posterior putamen (pPUT_rh; *χ* ^2^ = 8.11, p_FDR = 0.040). All four showed higher median QSM values in T2DM patients than in controls (Table 2). Detailed QSM values for both the T2DM and HC groups are provided in Table S12.

**TABLE 2:** Comparison of regional brain magnetic susceptibility values between the T2DM and HC groups.

Variables	Statistic	*p*	p*
aHIP_rh	0.805	0.371	0.495
pHIP_rh	3.063	0.082	0.175
lAMY_rh	1.006	0.317	0.441
mAMY_rh	2.098	0.149	0.238
THA_DP_rh	3.578	0.06	0.160
THA_VP_rh	4.616	**0.033**	0.132
THA_VA_rh	6.99	**0.009**	0.058
THA_DA_rh	8.853	**0.003**	**0.032**
NAc_shell_rh	6.528	**0.011**	0.059
NAc_core_rh	3.095	0.08	0.175
pGP_rh	0.006	0.939	0.939
aGP_rh	0.035	0.851	0.906
aPUT_rh	10.41	**0.001**	**0.016**
pPUT_rh	8.11	**0.005**	**0.040**
aCAU_rh	2.425	0.121	0.228
pCAU_rh	12.476	**< 0.001**	**0.016**
aHIP_lh	0.038	0.846	0.906
pHIP_lh	2.178	0.141	0.238
lAMY_lh	0.024	0.878	0.906
mAMY_lh	0.241	0.624	0.713
THA_DP_lh	1.537	0.216	0.329
THA_VP_lh	1.36	0.245	0.356
THA_VA_lh	3.314	0.07	0.172
THA_DA_lh	3.647	0.057	0.160
NAc_shell_lh	3.661	0.057	0.160
NAc_core_lh	0.482	0.488	0.601
pGP_lh	2.156	0.143	0.238
aGP_lh	0.317	0.574	0.680
aPUT_lh	3.92	0.049	0.160
pPUT_lh	2.455	0.119	0.228
aCAU_lh	0.698	0.404	0.517
pCAU_lh	5.254	**0.023**	0.105

Note: P*, FDR correction. Bold values indicate statistical significance at *p* < 0.05.

Abbreviations: aGP, anterior globus pallidus; aHIP, anterior hippocampus; aPUT, anterior putamen; lAMY, lateral amygdala; lh, left hemisphere; NAc‐shell, nucleus accumbens, shell; pCAU, posterior caudate; rh, right hemisphere; THA‐DA, dorsoanterior thalamus.

### 3.3 Association Between Magnetic Susceptibility Values and Cognitive Test

In the right anterior putamen (a‐PUT‐rh), three of the four Spearman partial correlations remained significant after Bonferroni correction (*α* = 0.05/16 = 0.003): with SDMT (*r* = −0.20, *p* = 0.0029), SCWT time (*r* = 0.21, *p* = 0.0019) and TMT time (*r* = 0.15, *p* = 0.0024), indicating that higher susceptibility is linked to slower processing speed and reduced executive function. The AVLT correlation (*r* = −0.14, *p* = 0.038) did not survive correction.

In the right posterior putamen (p‐PUT‐rh), only the SDMT association remained significant (*r* = −0.20, *p* = 0.0026); its relationships with AVLT (*p* = 0.042), SCWT (*p* = 0.0045) and TMT (*p* = 0.038) were no longer significant after adjustment.

For the right posterior caudate (p‐CAU‐rh), the negative correlation with SDMT persisted (*r* = −0.21, *p* = 0.0020), whereas its associations with SCWT (*p* = 0.011), TMT (*p* = 0.0098) and AVLT (*p* = 0.32) did not exceed the corrected threshold.

**FIGURE 3:**
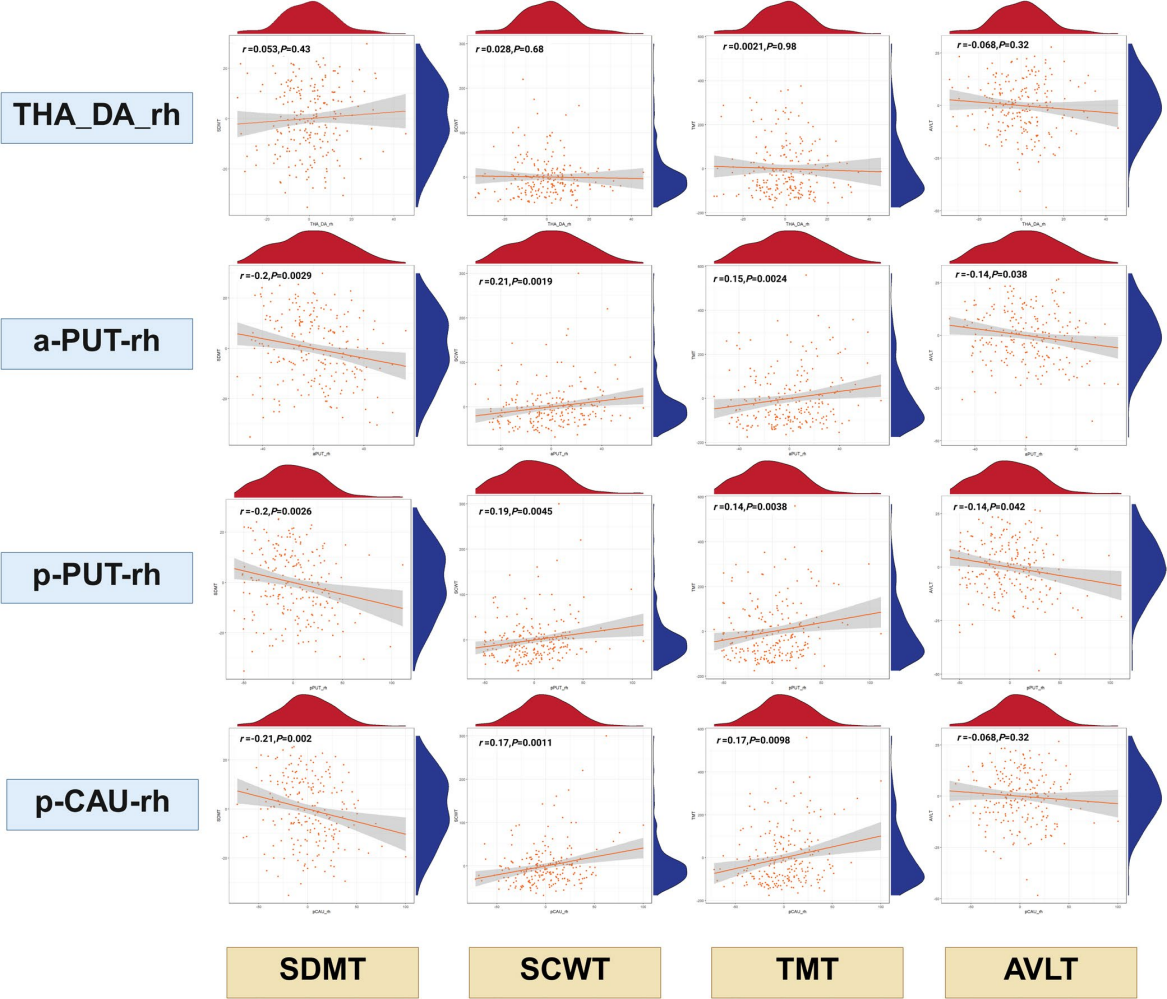
Correlations between subcortical nuclei magnetic susceptibility values and cognitive test scores. *p*‐values from Spearman correlations were Bonferroni‐corrected; the significance threshold was set at *p* < 0.05/16 = 0.003. Scatterplots with overlaid Spearman partial correlation lines; marginal density plots show the distribution of each variable. Spearman correlations are presented. The included cognitive tests are SCWT (Stroop Color‐Word Test, evaluating the ability to resolve color‐word interference), AVLT (Auditory Verbal Learning Test, measuring memory performance in auditory verbal tasks), TMT (Trail Making Test, highlighting motivational characteristics), and SDMT (Symbol Digit Modalities Test, assessing cognitive processing speed and symbol‐digit association skills). aPUT, anterior putamen; lh, left hemisphere; pPUT, posterior putamen; pCAU, posterior caudate; rh, right hemisphere; THA‐DA, dorsoanterior thalamus.

No significant correlations were observed between susceptibility in the right dorsoanterior thalamus (THA‐DA‐rh) and any cognitive test (Figure 3).

### 3.4 Nonlinear Relationship Between Magnetic Susceptibility Values and Metabolic Factors

**FIGURE 4:**
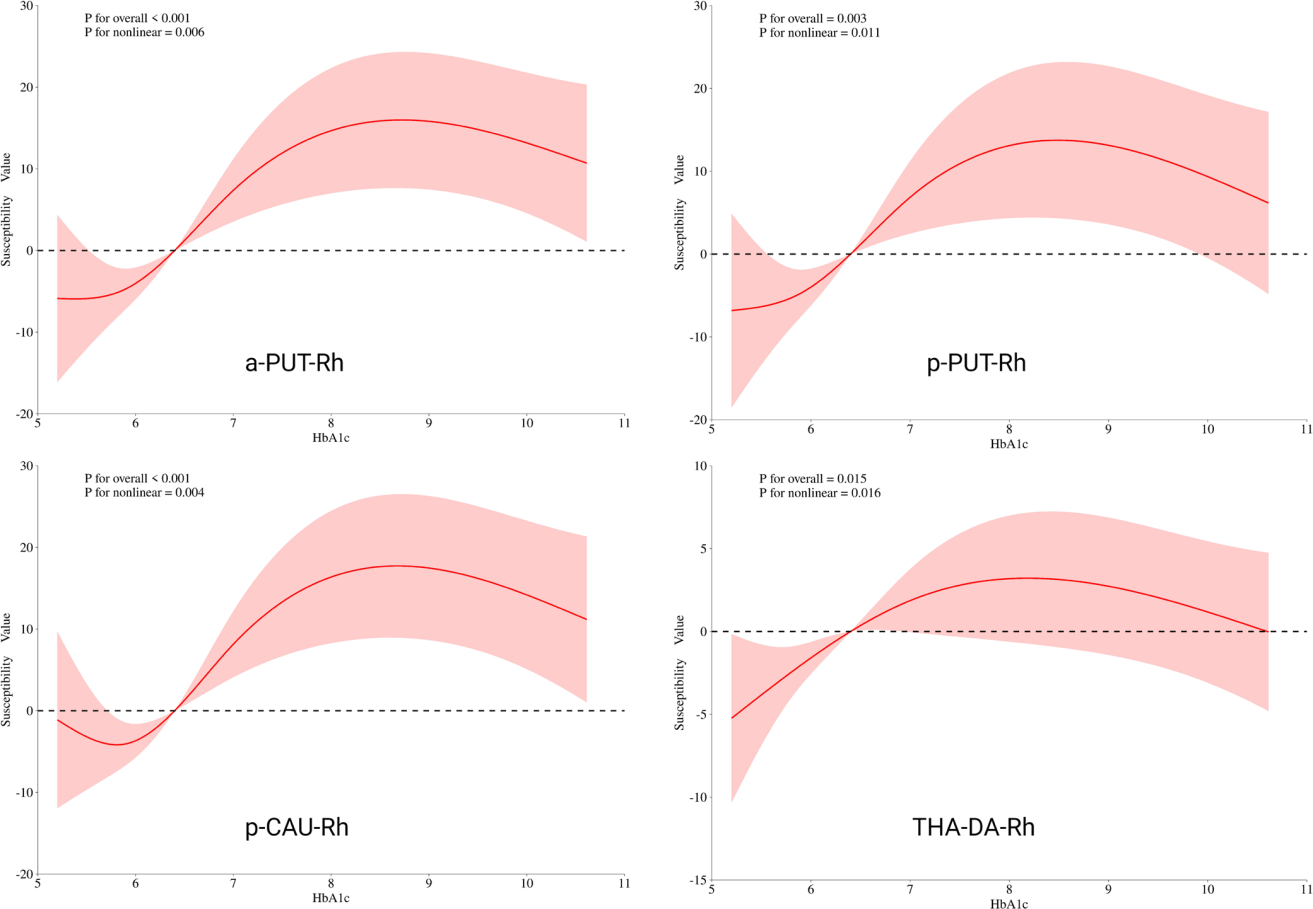
Non‐linear relationship curve between magnetic susceptibility values of subcortical nuclei and HbA1c. aPUT, anterior putamen; lh, left hemisphere; pCAU, posterior caudate; rh, right hemisphere; THA‐DA, dorsoanterior thalamus.

RCP analyses in the four ROIs that remained significant after FDR correction (THA_DA_rh, aPUT_rh, pPUT_rh and pCAU_rh) revealed significant overall associations between HbA1c and magnetic susceptibility values, indicating that fluctuations in HbA1c are linked to regional changes in brain tissue susceptibility. For the anterior and posterior putamen as well as the posterior caudate, magnetic susceptibility values show an initial decline at lower HbA1c levels, followed by an increase as HbA1c levels rise. In contrast, the thalamus shows a more gradual increase in magnetic susceptibility values, with a peak at moderate HbA1c levels before stabilizing (Figure 4).

Compared to HbA1c, FBG showed weaker associations with magnetic susceptibility values in the same nucleus. In aPUT_rh and pCAU_rh, RCS analysis revealed significant overall associations; however, no significant nonlinear trends were observed (*p* for nonlinear > 0.05). Similar results were found in the pPUT_rh, where the overall association was significant, but the relationship was predominantly linear. For THA_DA_rh, no significant association was detected.

### 3.5 Structural Equation Modeling of the Relationships Between Metabolic Factors, Magnetic Susceptibility Values, and Cognitive Function

To investigate the impact of general diabetes control on the magnetic susceptibility values of brain nuclei and cognitive function, we constructed a structural equation model (SEM). Given the complexity of the model, we included age as the sole covariate to account for potential confounding effects associated with age‐related changes in brain structure and cognition. Considering the similarities between AVLT and SDMT scores, we used three cognitive tests (SDMT, SCWT, and TMT) to create a latent variable representing cognitive ability. Furthermore, considering the lack of significant correlation between THA_DA_rh and cognitive function, as well as FBG and HbA1c, we excluded it from the SEM model.

#### 3.5.1 Model Fit and Evaluation

The model fit was assessed using several indices, including the chi‐square statistic (*χ* ^2^), root mean square error of approximation (RMSEA), Tucker–Lewis index (TLI), and comparative fit index (CFI). The fit statistics were as follows: *χ* ^2^ = 83.695, df = 31, *p* < 0.001, RMSEA = 0.068, CFI = 0.959, TLI = 0.940, and SRMR = 0.069, indicating an adequate fit to the data.

The *χ* ^2^/df ratio was 2.71, which is below the commonly accepted threshold of 3, suggesting a good model fit. Moreover, the RMSEA value of 0.068 is below the ideal threshold of 0.08, confirming that the error of approximation is within acceptable limits for complex models (Hu and Bentler 1999). Furthermore, both the CFI and TLI values exceeded the widely recommended threshold of 0.90, providing additional evidence that the model fit the data well.

#### 3.5.2 Measurement Error and Variance Estimates

The SEM model reveals significant relationships between various variables, with aPUT‐rh and pPUT‐rh showing high *R* ^2^ values of 0.865 and 0.864, respectively, indicating they are key predictors of magnetic susceptibility values. The SCWT, TMT, and SDMT tests explain 31.6%, 60.2%, and 89.9% of the variance in cognitive function, respectively. FBG and HbA1c squared contribute significantly to the model, with FBG explaining 38.9% of the variance in metabolic factors and HbA1c squared accounting for 44.7%. While magnetic susceptibility values has a strong standardized effect (0.950), its low *R* ^2^ of 0.050 suggests limited explanatory power in the model. Cognitive Function is explained by the model to the extent of 43.1%, reflecting its importance in the structure (Table 3).

**TABLE 3:** Measurement error and variance estimates.

Variable	Est	SE	*Z*	*p*	Std. (latent)	Std. (all)	*R* ^2^
pCAU_rh	0.368	0.051	7.238	**< 0.001**	0.368	0.372	0.628
aPUT_rh	0.133	0.039	3.388	0.001	0.133	0.135	0.865
pPUT_rh	0.134	0.026	5.085	**< 0.001**	0.134	0.136	0.864
SCWT	0.672	0.161	4.163	**< 0.001**	0.672	0.684	0.316
TMT	0.387	0.087	4.426	**< 0.001**	0.387	0.398	0.602
SDMT	0.096	0.050	1.934	0.053	0.096	0.101	0.899
FBG	0.608	0.117	5.191	**< 0.001**	0.608	0.611	0.389
HbA1c squared	2.209	0.673	3.281	0.001	2.209	0.553	0.447
Magnetic susceptibility values	0.590	0.089	6.659	**< 0.001**	0.950	0.950	0.050
Cognitive function	0.176	0.049	3.609	**< 0.001**	0.569	0.569	0.431
Metabolic factor	0.388	0.158	2.452	0.014	1.000	1.000	

*Note:* Bold values indicate statistical significance at *p* < 0.05.

Abbreviations: Est (estimate), the estimated value of each path or parameter, reflecting the strength of the relationship between variables; *p*‐value, the *p*‐value for testing the significance of the path coefficient, with values below 0.05 indicating statistical significance; *R* ^2^, the *R*‐squared value, representing the proportion of variance in the dependent variable explained by the model; SE (standard error), the standard error of the estimate, indicating the precision of the estimated value; Std. (all), the standardized coefficient for all variables (latent and observed), representing the effect of each predictor on the outcome; Std. (latent), the standardized coefficient for latent variables, indicating the impact of the latent variable on the outcome; *Z*, the *Z*‐value, calculated as the estimate divided by the standard error, used to test the significance of the estimate.

#### 3.5.3 Impact of Metabolic Factors on Magnetic Susceptibility Values and Cognitive Function

In our SEM measurement model, magnetic susceptibility values are a latent factor measured by three observed variables: pCAU_rh (*λ* = 1.000, fixed for scale identification), aPUT_rh (*λ* = 0.930) and pPUT_rh (*λ* = 0.930), indicating that these regional QSM values strongly reflect the iron‐deposition construct (Table 4). Cognitive function is similarly measured by TMT (*λ* = 0.776), SCWT (*λ* = 0.562) and SDMT (*λ* = 1.668), with SDMT as the most robust indicator. Finally, the Metabolic Factor latent variable is indicated by FBG (*λ* = 0.624), HbA1c (*λ* = 1.115) and HbA1c^2^ (*λ* = 0.669). In SEM, factor loadings represent the strength of the relationship between each observed indicator and its latent factor. Higher loadings mean that an indicator captures the underlying construct more accurately and meaningfully.

**TABLE 4:** Path coefficients table.

Lhs	Rhs	Est	SE	*Z*	*p*	Lower 95% CI	Upper 95% CI	Std. (all)
Magnetic susceptibility values	pCAU rh	1.000^a^	0.000			1.000	1.000	0.792
Magnetic susceptibility values	aPUT rh	1.172	0.091	12.924	**< 0.001**	1.008	1.361	0.930
Magnetic susceptibility values	pPUT rh	1.172	0.095	12.334	**< 0.001**	0.998	1.374	0.930
Cognitive function	SCWT	1.000^a^	0.000			1.000	1.000	0.562
Cognitive function	TMT	1.372	0.176	7.805	**< 0.001**	1.088	1.790	0.776
Cognitive function	SDMT	1.668	0.274	6.087	**< 0.001**	1.236	2.306	0.948
Metabolic factor	FBG	1.000^a^	0.000			1.000	1.000	0.624
Metabolic factor	HbA1c	1.786	0.618	2.892	0.004	1.145	3.389	1.115
Metabolic factor	HbA1c squared	2.148	0.509	4.218	**< 0.001**	1.195	3.176	0.669
Magnetic susceptibility values	Metabolic factor	0.224	0.086	2.589	0.010	0.070	0.409	0.177
Magnetic susceptibility values	Age	0.108	0.058	1.863	0.063	−0.007	0.230	0.137
Cognitive function	Magnetic susceptibility values	−0.249	0.056	−4.482	**< 0.001**	−0.380	−0.162	−0.353
Cognitive function	Metabolic factor	−0.086	0.064	−1.342	0.180	−0.226	0.030	−0.097
Cognitive function	Age	−0.353	0.055	−6.409	**< 0.001**	−0.465	−0.248	−0.633

*Note:* Bold values indicate statistical significance at *p* < 0.05.

Abbreviations: aPUT, anterior putamen; CI, confidence interval; Est, estimate; lh, left hemisphere; Lhs, left‐hand side; pCAU, posterior caudate; rh, right hemisphere; Rhs, right‐hand side; SE, standard error; Std.Est, standardized estimate.

^a^ FBG, SCWT, and pCAU_rh are fixed at 1.000 for model identification purposes. The standard error is zero because these parameters are fixed.

**FIGURE 5:**
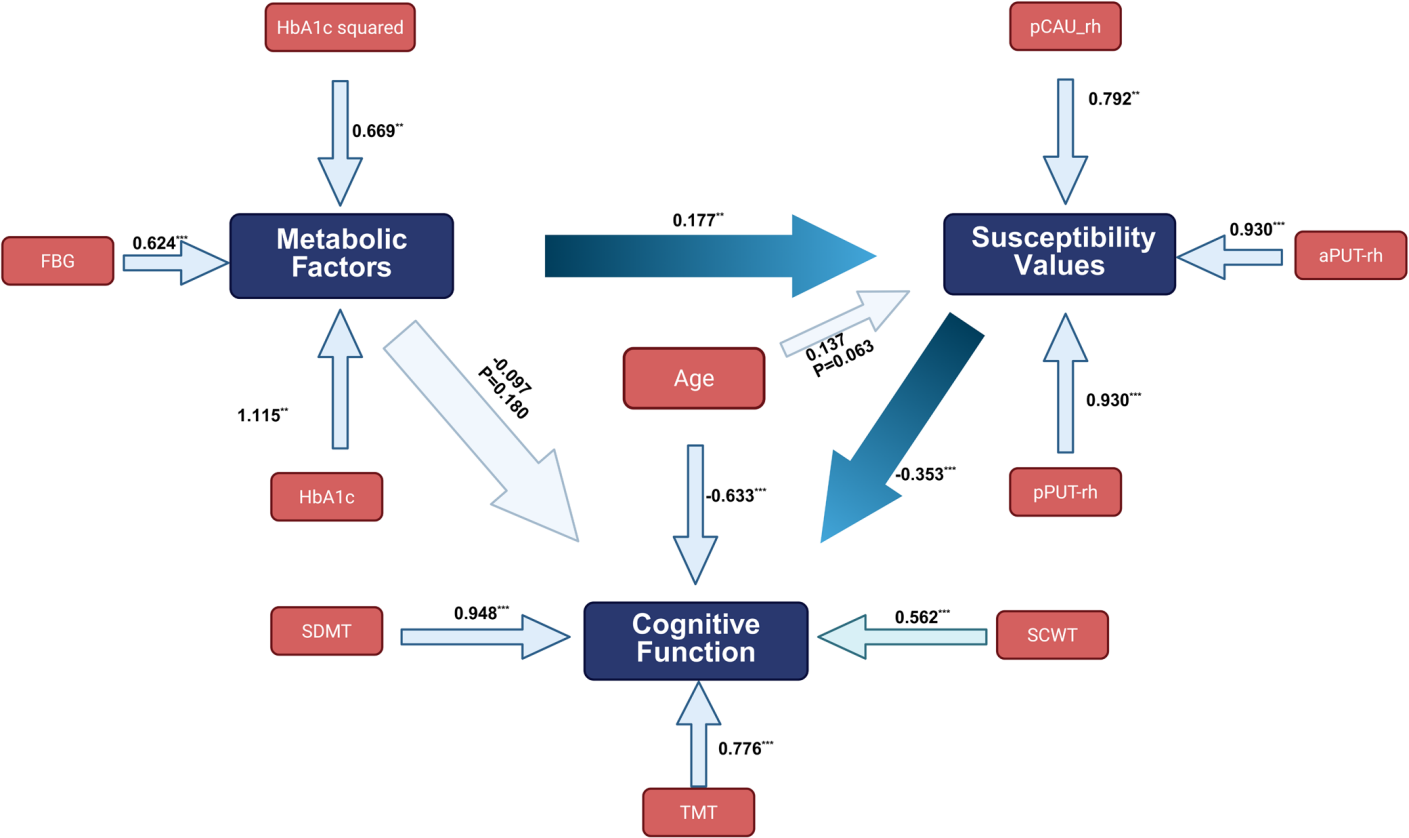
Structural equation model of cognitive, metabolic, and magnetic susceptibility values interactions. The relationships among metabolic changes, magnetic susceptibility values of subcortical nuclei, and cognitive function are depicted in the SEM. The term “HbA1c squared” represents the squared value of HbA1c (HbA1c * HbA1c). Significant paths are marked as follows: *p < 0.05, **p < 0.01, and ***p < 0.001, indicating the strength of the statistical associations.

The relationships between the constructs in the SEM model indicate that metabolic factor positively influences magnetic susceptibility values (estimate = 0.224), suggesting that higher metabolic factors are associated with higher magnetic susceptibility values. On the other hand, cognitive function is negatively influenced by both magnetic susceptibility values (−0.249) and age (−0.353), suggesting that increased magnetic susceptibility values and older age are linked to poorer cognitive performance. Interestingly, the relationship between the metabolic factor and cognitive function is not significant (*p* = 0.180), indicating that metabolic factors have a lesser impact on cognitive function compared to brain susceptibility and age (Figure 5). Although the direct path from Metabolic Factor to Cognitive Function was not significant (*β* = −0.086, *p* = 0.180), the total effect was significant (*β* = −0.142, *p* = 0.037), demonstrating that metabolic dysfunction impacts cognition primarily through altered brain iron deposition (Table 5).

**TABLE 5:** Indirect and total effects table.

Effect type		Est	SE	*Z*	*p*	Lower 95% CI	Upper 95% CI	Std.Est
Indirect effect	Metabolic factor → magnetic susceptibility values → cognitive function	−0.056	0.024	−2.362	0.018	−0.105	−0.016	−0.062
Total effect	Metabolic factor → cognitive function	−0.142	0.068	−2.087	0.037	−0.296	−0.028	−0.159

Abbreviations: Lower 95% CI/Upper 95% CI, the lower and upper bounds of the confidence interval for the effect; Est (estimate), the estimated value of the effect, indicating the magnitude and direction of the relationship; *p*‐value, the *p*‐value for testing the significance of the effect; SE (standard error), the standard error of the estimated effect, measuring the precision of the estimate; Std.Est (standardized estimate), the standardized version of the effect size; *Z*, the *Z*‐value, calculated as the estimate divided by the standard error, used to assess the statistical significance of the effect.

To address the possibility of reverse causation, we conducted a supplementary analysis by adding a reverse path from cognitive function to metabolic factors (Metabolic Factor ~ Cognitive Function) (Table S4). In this supplementary model, the estimated coefficient for the reverse path was −0.093 (SE = 0.054, *Z* = −1.707, *p* = 0.088), which did not reach statistical significance. This finding indicates that although there is a slight trend toward a reverse influence, its effect is relatively weak compared to the significant impact of metabolic dysregulation on brain iron deposition and, consequently, on cognitive function (Table S5).

#### 3.5.4 Sensitivity Analysis

To test the robustness of our SEM, we added Triglycerides (TG) and High‐density lipoprotein cholesterol (HDL) as observed indicators of the Metabolic Factor (Table S6). This expanded model showed poorer fit (CFI = 0.89, RMSEA = 0.12) compared to the original (CFI = 0.96, RMSEA = 0.068), suggesting that lipid markers introduce noise rather than clarity (Table S8). Importantly, the mediation remains robust, with the indirect effect through magnetic susceptibility at −0.054 (*p* = 0.024) and the total effect at −0.133 (*p* = 0.034), which are almost identical to the original model's results. This confirms that blood‐glucose metrics are the dominant metabolic drivers of brain iron deposition and cognitive decline in our cohort (Table S7).

### 3.6 Polynomial Regression Analysis of Factors Influencing Magnetic Susceptibility Values

All multivariate regression models simultaneously included the following independent variables: age, sex, BMI, FBG, HbA1c and its squared term (HbA1c^2^), as well as the clinical covariates—T2DM diagnosis, hypertension, hyperlipidemia, smoking status, and alcohol status.

For the a‐PUT‐rh, HbA1c was the strongest predictor, showing a significant positive association in both univariate (*β* = 3.25, SE = 0.94, *p* < 0.001) and multivariate analyses (*β* = 23.41, SE = 6.29, *p* < 0.001). The squared term of HbA1c also indicated a significant nonlinear effect (*β* = −1.21, SE = 0.37, *p* = 0.001), suggesting a curvilinear relationship between HbA1c and magnetic susceptibility values. FBG was a significant predictor in the univariate analysis (*β* = 2.44, SE = 0.71, *p* < 0.001), though it was not retained in the multivariate model. Age (*β* = 0.47, SE = 0.22, *p* = 0.031) and diabetes diagnosis (*β* = 10.49, SE = 3.51, *p* = 0.003) were also significant predictors in the multivariate model, emphasizing the influence of both age‐related changes and metabolic disorders (Table S1).

In the p‐PUT‐rh, age (*β* = 0.62, SE = 0.25, *p* = 0.013), FBG (*β* = 2.42, SE = 0.79, *p* = 0.002) and T2DM diagnosis (*β* = 11.12, SE = 3.90, *p* = 0.005) were significant multivariate predictors, while HbA1c was only significant univariately (*β* =2.45, SE = 1.07, *p* = 0.023) with a non‐significant squared term (Table S2).

For the p‐CAU‐rh, HbA1c was again the most prominent predictor, with a strong positive association in the multivariate model (*β* = 26.80, SE = 6.62, *p* < 0.001) and a significant nonlinear effect from its squared term (*β* = −1.40, SE = 0.39, *p* < 0.001). Gender was significant in the multivariate model (*β* = −8.88, SE = 3.69, *p* = 0.017), with males exhibiting higher magnetic susceptibility values. T2DM diagnosis (*β* = 12.83, SE = 3.63, *p* < 0.001) and hyperlipidemia (*β* = 8.67, SE = 3.71, *p* = 0.020) were also significant, indicating the contribution of systemic metabolic health, whereas FBG was only significant in univariate analysis (*β* = 2.76, SE = 0.74, *p* < 0.001) (Table S3).

### 3.7 Moderating Effects of Age on the Relationship Between Blood Glucose Dysfunction and Cognitive Function

To further examine whether age moderates the impact of blood glucose dysfunction on cognitive function, we conducted a series of traditional linear regression analyses, which included interaction terms between age and blood glucose measures (HbA1c and FBG). In the TMT model, the multivariate analysis showed that the Age × HbA1c interaction was significant (*β* = 0.24, *p* = 0.011) and the Age × FBG interaction was also significant (*β* = −0.15, *p* = 0.043) (Table S9). In the SCWT model, the multivariate analysis revealed that both the Age × HbA1c interaction (*β* = 0.11, *p* = 0.002) and the Age × FBG interaction (*β* = 0.18, *p* = 0.007) were statistically significant (Table S10). Similarly, in the SDMT model, the multivariate analysis demonstrated a robust negative association for the Age × HbA1c interaction (*β* = −0.16, *p* < 0.001) (Table S11). Moreover, in all models, the iron deposition in the pCAU‐rh was significantly associated with cognitive performance (*p* < 0.05).

## 4 Discussion

In this study, we assessed the impact of changes in the QSM values of specific subcortical nuclei in T2DM patients on cognition during the progression of diabetes. Our research provides a detailed pattern of iron homeostasis in subcortical nuclei between T2DM patients and HCs and preliminarily confirms the critical role of iron deposition in the cognitive damage caused by blood glucose fluctuations. Many previous studies have confirmed that QSM technology outperforms traditional imaging techniques in identifying brain damage related to T2DM and its impact on cognition (Zhang et al. 2019). Given that the CAU and PUT may be potential candidates for identifying early cognitive impairment in T2DM patients, our findings provide important insights into the pathogenesis of TDACD and offer key information for the early prevention of poorer cognitive performance.

High QSM values in the principal basal ganglia nuclei are particularly meaningful because these regions both accumulate non‐heme iron selectively and subserve critical motor and executive functions. Yang et al. demonstrated that T2DM patients exhibit markedly elevated susceptibility in the CAU and PUT compared with controls, and that these elevations correlate with slower processing speed and executive deficits (Yang et al. 2018). A recent meta‐analysis pooling six QSM studies confirmed moderate‐to‐strong increases in the PUT and CAU in T2DM versus controls, whereas regions like the globus pallidus and substantia nigra showed weaker or nonsignificant changes (Mohammadi et al. 2024). These convergent findings underscore that iron‐driven susceptibility shifts in the dorsal striatum not only mark early neuropathology but also track the severity of cognitive impairment in diabetes.

To enhance specificity for iron and disambiguate demyelination or vascular confounds, several groups have combined QSM with complementary quantitative MRI measures. Li et al. used QSM alongside susceptibility‐weighted angiography to map heterogeneous iron distributions in the extrapyramidal system of Parkinson's patients with T2DM, revealing that regional susceptibility metrics align closely with clinical severity (Li et al. 2022). Moreover, Ni et al. integrated QSM‐derived iron maps with arterial spin‐labeling–derived cerebral blood flow to quantify neurovascular coupling in T2DM patients, finding that reduced coupling between susceptibility and perfusion deficits predicts worse memory and executive scores (Ni et al. 2023).

In this research, we explored the cross‐sectional association between glycemic variability and magnetic susceptibility values of subcortical nuclei, revealing its close link to brain iron homeostasis. The study confirmed that elevated HbA1c levels were significantly positively correlated with magnetic susceptibility values in subcortical nuclei, such as the CAU and PUT, suggesting a key role of metabolic abnormalities in brain microstructural changes. Iron is an essential cofactor involved in oxygen binding and transport, energy, and material metabolism, influencing oxygen transport, cell growth regulation, electron transfer, and DNA synthesis (Apostolakis and Kypraiou 2017). However, disruptions in iron homeostasis can result in the excessive production of reactive oxygen species, leading to oxidative stress and eventual cell apoptosis (Galaris et al. 2019). Hyperglycemia has been shown to exacerbate the permeability of the blood–brain barrier (BBB), leading to increased vascular permeability and edema (Banks and Rhea 2021; Rhea and Banks 2019). BBB disruption allows toxic plasma components to leak into the brain parenchyma, causing local damage and leading to cognitive dysfunction (Banks 2020; Uchida, Kan, Sakurai, et al. 2023).

Fluctuating glycemia exerts a dual assault on iron homeostasis by promoting both barrier breakdown and oxidative‐inflammatory cascades that impair iron metabolism and clearance. Sui et al. reported that T2DM patients exhibit increased basal ganglia susceptibility values driven more by age and diabetes duration than by neuropathy status, linking higher iron deposition to poorer motor and cognitive performance (Sui et al. 2025). Dysglycemia‐induced endothelial dysfunction also triggers microglial activation and upregulation of inflammatory mediators in diabetic rodent models, paralleling enhanced BBB leakage and memory deficits. Intriguingly, Zhao et al. found that T2DM patients with mild cognitive impairment display a localized decrease in QSM‐measured iron in the temporal cortex—perhaps reflecting a compensatory response—while those with normal cognition do not, suggesting dynamic iron redistribution in early diabetic neurodegeneration (Zhao et al. 2025). Recent studies in other neurological disorders, such as CADASIL, further support the role of BBB disruption in iron accumulation and cognitive impairment. For instance, a cross‐sectional study in CADASIL patients demonstrated that voxel‐based QSM analysis revealed significantly higher magnetic susceptibility values in regions like the PUT and CAU nucleus in symptomatic individuals compared to asymptomatic carriers and HCs. These elevated QSM values were positively correlated with regional BBB permeability and inversely correlated with cognitive performance (Uchida et al. 2020). Additionally, oxidative stress triggered by insulin resistance may interfere with brain iron metabolism, leading to excessive iron deposition, particularly in vulnerable regions such as the basal ganglia and prefrontal cortex (Blasco et al. 2014). This accumulation of iron further amplifies oxidative stress, potentially causing progressive neuronal damage and poorer cognitive performance (Sobieska et al. 2024).

The dorsal striatum, also referred to as the new striatum, is composed of the CAU nucleus and PUT (Haber 2016). The ventral striatum is the ventral convergence of the CAU and PUT, merging and including the striatal portions of the nucleus accumbens and olfactory tubercle. In our study, we identified the right CAU nucleus and PUT as the nuclei most significantly influenced by HbA1c levels, with both showing significant correlations with various cognitive test scores. Supporting these findings, a large‐scale study from the UK Biobank revealed that T2DM diagnosis was significantly associated with gray matter atrophy, particularly in regions including the ventral striatum, cerebellum, and CAU nucleus (Antal et al. 2022). This atrophy was also accompanied by evidence of brain activity reorganization, further highlighting the impact of metabolic dysregulation on brain structure and function. Similarly, a data‐driven cohort study employing machine learning algorithms identified a negative correlation between the IR and the volumes of the hippocampus, amygdala, and PUT (Gentreau et al. 2022). Our results complement these findings by highlighting the critical mechanistic role of elevated blood glucose levels in the dorsal striatum. Given their significant indirect effects on glucose‐induced cognitive dysfunction, the CAU nucleus and PUT may represent key targets for interventions aimed at mitigating cognitive impairments in T2DM patients. These findings provide new insights into early prediction and intervention for TDACD.

While our study focused on subcortical iron dysregulation, recent research has underscored the importance of cortical iron accumulation in age‐related network dysfunction and cognitive decline. For example, one recent QSM study reported that higher cortical magnetic susceptibility values in older adults are linked to reduced task‐based functional connectivity within the frontoparietal working‐memory network and worse N‐Back performance (Zachariou et al. 2020). An ultra‐high‐field QSM investigation then revealed marked age‐related iron increases in layer 5 of the primary motor cortex, highlighting depth‐dependent cortical vulnerability (Northall et al. 2023). More recently, a lifespan analysis using column‐based, depthwise QSM showed that elevated superficial cortical iron predicts lower resting‐state system segregation and mediates age‐related declines in fluid cognition (Merenstein et al. 2025). These findings suggest that future studies should expand our QSM analyses to cortical regions by mapping iron across both layers and depths and by examining functional connectivity during cognitive tasks and at rest, to clarify how iron dysregulation throughout gray matter is associated with poorer cognitive performance in T2DM.

However, our study has several limitations. While we compared the QSM values of subcortical nuclei in different T2DM patients, we did not investigate how these changes vary across different stages of the disease. Given the high prevalence of T2DM in older populations, we excluded participants under 40 years of age from the analysis to mitigate the influence of age on nucleus magnetic susceptibility values (Izzo et al. 2021). However, our study population was still primarily older adults, which is an unavoidable situation and may reduce the generalizability of our findings.

Future research will focus on investigating the interactions between subcortical nuclei during the progression of TDACD and examining the link between iron homeostasis imbalance and cognitive impairment. Ultimately, our goal is to develop an early prediction model based on QSM to assess the risk of TDACD, enabling precise disease prediction and timely intervention.

## 5 Conclusion

This study highlights the intricate relationship between T2DM and cognitive decline, emphasizing the impact of blood glucose fluctuations on brain iron homeostasis. These findings suggest that T2DM patients are at increased risk of cognitive dysfunction due to disruptions in brain iron levels and glucose regulation. By measuring changes in magnetic susceptibility values in subcortical nuclei, we identified key brain regions where iron metabolism is disrupted, with these changes further exacerbated by blood glucose fluctuations. These insights are critical for understanding how metabolic dysregulation in T2DM contributes to neurodegeneration.

## Ethics Statement

All study procedures were approved by the Ethical Committee of the Institutional Review Board (IRB) of Shandong Institute of Medical Imaging (2019‐002).

## Consent

All authors approved the final manuscript and the submission to this journal.

## Conflicts of Interest

The authors declare no conflicts of interest.

## Supporting information


Data S1.

## Data Availability

The data that support the findings of this study are available on request from the corresponding author. The data are not publicly available due to privacy or ethical restrictions.
