# Supplementary material for: The Role of Iron Homeostasis Imbalance in T2DM‐Associated Cognitive Dysfunction: A Prospective Cohort Study Utilizing Quantitative Susceptibility Mapping
Source: Hum Brain Mapp. 2025 Jun 18;46(9):e70263. doi: 10.1002/hbm.70263 (PMC12175199; doi:10.1002/hbm.70263)
Supplement: Supplementary file 1 — Data S1. [file HBM-46-e70263-s001.docx]

**Table S1** : Polynomial regression analysis of aPUT_rh susceptibility balues

| Variables | Univariate | | | | |  | Multivariate | | | | |
| --- | --- | --- | --- | --- | --- | --- | --- | --- | --- | --- | --- |
|  | β | S.E | t | *P* | β (95%CI) |  | β | S.E | t | *P* | β (95%CI) |
| Age | 0.62 | 0.22 | 2.79 | **0.006** | 0.62 (0.19 ~ 1.06) |  | 0.47 | 0.22 | 2.17 | **0.031** | 0.47 (0.05 ~ 0.90) |
| HbA1c | 3.25 | 0.94 | 3.45 | **<.001** | 3.25 (1.40 ~ 5.09) |  | 23.41 | 6.29 | 3.72 | **<.001** | 23.41 (11.09 ~ 35.73) |
| HbA1c squared | 0.16 | 0.06 | 2.89 | **0.004** | 0.16 (0.05 ~ 0.27) |  | -1.21 | 0.37 | -3.28 | **0.001** | -1.21 (-1.93 ~ -0.49) |
| FBG | 2.44 | 0.71 | 3.46 | **<.001** | 2.44 (1.06 ~ 3.82) |  |  |  |  |  |  |
| BMI | 0.95 | 0.57 | 1.68 | 0.094 | 0.95 (-0.16 ~ 2.06) |  |  |  |  |  |  |
| Gender |  |  |  |  |  |  |  |  |  |  |  |
| Male |  |  |  |  | 0.00 (Reference) |  |  |  |  |  |  |
| Female | -2.23 | 3.55 | -0.63 | 0.532 | -2.23 (-9.19 ~ 4.74) |  |  |  |  |  |  |
| Hypertension |  |  |  |  |  |  |  |  |  |  |  |
| No |  |  |  |  | 0.00 (Reference) |  |  |  |  |  |  |
| Yes | 8.07 | 3.56 | 2.27 | **0.024** | 8.07 (1.09 ~ 15.05) |  |  |  |  |  |  |
| Hyperlipidaemia |  |  |  |  |  |  |  |  |  |  |  |
| No |  |  |  |  | 0.00 (Reference) |  |  |  |  |  |  |
| Yes | 5.14 | 3.56 | 1.45 | 0.150 | 5.14 (-1.83 ~ 12.12) |  |  |  |  |  |  |
| T2DM |  |  |  |  |  |  |  |  |  |  |  |
| No |  |  |  |  | 0.00 (Reference) |  |  |  |  |  |  |
| Yes | 11.22 | 3.48 | 3.23 | **0.001** | 11.22 (4.40 ~ 18.03) |  |  |  |  |  |  |
| Smoke |  |  |  |  |  |  |  |  |  |  |  |
| No |  |  |  |  | 0.00 (Reference) |  |  |  |  |  |  |
| Yes | 2.13 | 4.11 | 0.52 | 0.604 | 2.13 (-5.93 ~ 10.19) |  |  |  |  |  |  |
| Drink |  |  |  |  |  |  |  |  |  |  |  |
| No |  |  |  |  | 0.00 (Reference) |  |  |  |  |  |  |
| Yes | -2.37 | 3.77 | -0.63 | 0.530 | -2.37 (-9.75 ~ 5.01) |  |  |  |  |  |  |

CI: Confidence Interval

**Table S2** : Polynomial regression analysis of pPUT_rh susceptibility balues

| Variables | Univariate | | | | |  | Multivariate | | | | |
| --- | --- | --- | --- | --- | --- | --- | --- | --- | --- | --- | --- |
|  | β | S.E | t | *P* | β (95%CI) |  | β | S.E | t | *P* | β (95%CI) |
| Age | 0.58 | 0.25 | 2.31 | **0.022** | 0.58 (0.09 ~ 1.07) |  | 0.62 | 0.25 | 2.51 | **0.013** | 0.62 (0.14 ~ 1.11) |
| HbA1c | 2.45 | 1.07 | 2.30 | **0.023** | 2.45 (0.36 ~ 4.54) |  |  |  |  |  |  |
| HbA1c squared | 0.11 | 0.06 | 1.77 | 0.079 | 0.11 (-0.01 ~ 0.23) |  |  |  |  |  |  |
| FBG | 2.31 | 0.79 | 2.91 | **0.004** | 2.31 (0.76 ~ 3.87) |  | 2.42 | 0.79 | 3.08 | **0.002** | 2.42 (0.88 ~ 3.96) |
| BMI | 0.73 | 0.63 | 1.15 | 0.252 | 0.73 (-0.51 ~ 1.97) |  |  |  |  |  |  |
| Gender |  |  |  |  |  |  |  |  |  |  |  |
| Male |  |  |  |  | 0.00 (Reference) |  |  |  |  |  |  |
| Female | -3.68 | 3.97 | -0.93 | 0.355 | -3.68 (-11.46 ~ 4.10) |  |  |  |  |  |  |
| Hypertension |  |  |  |  |  |  |  |  |  |  |  |
| No |  |  |  |  | 0.00 (Reference) |  |  |  |  |  |  |
| Yes | 5.85 | 4.01 | 1.46 | 0.146 | 5.85 (-2.01 ~ 13.70) |  |  |  |  |  |  |
| Hyperlipidaemia |  |  |  |  |  |  |  |  |  |  |  |
| No |  |  |  |  | 0.00 (Reference) |  |  |  |  |  |  |
| Yes | 3.56 | 3.99 | 0.89 | 0.373 | 3.56 (-4.26 ~ 11.37) |  |  |  |  |  |  |
| T2DM |  |  |  |  |  |  |  |  |  |  |  |
| No |  |  |  |  | 0.00 (Reference) |  |  |  |  |  |  |
| Yes | 11.12 | 3.90 | 2.85 | **0.005** | 11.12 (3.46 ~ 18.77) |  |  |  |  |  |  |
| Smoke |  |  |  |  |  |  |  |  |  |  |  |
| No |  |  |  |  | 0.00 (Reference) |  |  |  |  |  |  |
| Yes | 7.45 | 4.57 | 1.63 | 0.104 | 7.45 (-1.51 ~ 16.41) |  |  |  |  |  |  |
| Drink |  |  |  |  |  |  |  |  |  |  |  |
| No |  |  |  |  | 0.00 (Reference) |  |  |  |  |  |  |
| Yes | -0.01 | 4.21 | -0.00 | 0.997 | -0.01 (-8.27 ~ 8.24) |  |  |  |  |  |  |

CI: Confidence Interval

**Table S3** : Polynomial regression analysis of pCAU_rh susceptibility balues

| Variables | Univariate | | | | |  | Multivariate | | | | |
| --- | --- | --- | --- | --- | --- | --- | --- | --- | --- | --- | --- |
|  | β | S.E | t | *P* | β (95%CI) |  | β | S.E | t | *P* | β (95%CI) |
| Age | 0.40 | 0.24 | 1.70 | 0.091 | 0.40 (-0.06 ~ 0.87) |  |  |  |  |  |  |
| HbA1c | 3.12 | 0.99 | 3.14 | **0.002** | 3.12 (1.17 ~ 5.07) |  | 26.80 | 6.62 | 4.05 | **<.001** | 26.80 (13.82 ~ 39.79) |
| HbA1c squared | 0.15 | 0.06 | 2.58 | **0.011** | 0.15 (0.04 ~ 0.27) |  | -1.40 | 0.39 | -3.61 | **<.001** | -1.40 (-2.16 ~ -0.64) |
| FBG | 2.76 | 0.74 | 3.74 | **<.001** | 2.76 (1.31 ~ 4.20) |  |  |  |  |  |  |
| BMI | 1.03 | 0.59 | 1.73 | 0.085 | 1.03 (-0.14 ~ 2.19) |  |  |  |  |  |  |
| Gender |  |  |  |  |  |  |  |  |  |  |  |
| Male |  |  |  |  | 0.00 (Reference) |  |  |  |  |  |  |
| Female | -8.88 | 3.69 | -2.41 | **0.017** | -8.88 (-16.10 ~ -1.65) |  |  |  |  |  |  |
| Hypertension |  |  |  |  |  |  |  |  |  |  |  |
| No |  |  |  |  | 0.00 (Reference) |  |  |  |  |  |  |
| Yes | 7.22 | 3.75 | 1.92 | 0.056 | 7.22 (-0.13 ~ 14.57) |  |  |  |  |  |  |
| Hyperlipidaemia |  |  |  |  |  |  |  |  |  |  |  |
| No |  |  |  |  | 0.00 (Reference) |  |  |  |  |  |  |
| Yes | 8.67 | 3.71 | 2.34 | **0.020** | 8.67 (1.41 ~ 15.94) |  |  |  |  |  |  |
| T2DM |  |  |  |  |  |  |  |  |  |  |  |
| No |  |  |  |  | 0.00 (Reference) |  |  |  |  |  |  |
| Yes | 12.83 | 3.63 | 3.53 | **<.001** | 12.83 (5.70 ~ 19.95) |  |  |  |  |  |  |
| Smoke |  |  |  |  |  |  |  |  |  |  |  |
| No |  |  |  |  | 0.00 (Reference) |  |  |  |  |  |  |
| Yes | 8.66 | 4.28 | 2.02 | **0.044** | 8.66 (0.27 ~ 17.05) |  |  |  |  |  |  |
| Drink |  |  |  |  |  |  |  |  |  |  |  |
| No |  |  |  |  | 0.00 (Reference) |  |  |  |  |  |  |
| Yes | 4.66 | 3.94 | 1.18 | 0.239 | 4.66 (-3.08 ~ 12.39) |  |  |  |  |  |  |
| CI: Confidence Interval | | | | | | | | | | | |

**Table S4** : Alternative model with a reversed path from cognitive function to the metabolic factor Sensitivity analysis model

| Lhs | Rhs | Est | SE | Z | P-value | CI.lower | CI.upper | Std. (All) |
| --- | --- | --- | --- | --- | --- | --- | --- | --- |
| Susceptibility Values | pCAU rh | 1.000^*^ | 0.000 |  | . | 1.000 | 1.000 | 0.794 |
| Susceptibility Values | aPUT rh | 1.172 | 0.091 | 12.931 | <.001 | 1.010 | 1.381 | 0.931 |
| Susceptibility Values | pPUT rh | 1.172 | 0.095 | 12.383 | <.001 | 1.003 | 1.380 | 0.930 |
| Cognitive Function | SCWT | 1.000^*^ | 0.000 |  |  | 1.000 | 1.000 | 0.571 |
| Cognitive Function | TMT | 1.372 | 0.178 | 7.725 | <.001 | 1.087 | 1.812 | 0.783 |
| Cognitive Function | SDMT | 1.664 | 0.278 | 5.996 | <.001 | 1.220 | 2.365 | 0.950 |
| Metabolic Factor | FBG | 1.000^*^ | 0.000 |  |  | 1.000 | 1.000 | 0.601 |
| Metabolic Factor | HbA1c | 1.908 | 0.688 | 2.772 | 0.006 | 1.161 | 3.734 | 1.148 |
| Metabolic Factor | HbA1c squared | 2.153 | 0.520 | 4.143 | <.001 | 1.225 | 3.281 | 0.646 |
| Susceptibility Values | Metabolic Factor | 0.192 | 0.085 | 2.254 | 0.024 | 0.029 | 0.366 | 0.146 |
| Susceptibility Values | Age | 0.119 | 0.055 | 2.167 | 0.030 | 0.015 | 0.234 | 0.150 |
| Cognitive Function | Susceptibility Values | -0.252 | 0.056 | -4.462 | <.001 | -0.383 | -0.165 | -0.350 |
| Cognitive Function | Metabolic Factor | 0.012 | 0.054 | 0.221 | 0.825 | -0.093 | 0.133 | 0.013 |
| Cognitive Function | Age | -0.365 | 0.060 | -6.134 | <.001 | -0.488 | -0.250 | -0.640 |
| Metabolic Factor | Cognitive Function | -0.093 | 0.054 | -1.707 | 0.088 | -0.219 | -0.009 | -0.155 |

**Abbreviation:** aPUT: Anterior putamen, pCAU: Posterior caudate, rh: Right hemisphere, lh: Left hemisphere, Lhs: Left-Hand Side, Rhs:Right-Hand Side, Est:Estimate, SE:Standard Error, Std.Est:Standardized Estimate, CI: Confidence Interval.

^*^ FBG, SCWT, and pCAU_rh are fixed at 1.000 for model identification purposes. The standard error is zero because these parameters are fixed.

**Table S5** : Direct and indirect effects of the alternative model

| **Effect Type** |  | **Est** | **Se** | **Z** | **P-value** | **CI.lower** | **CI.upper** | **Std.Est** |
| --- | --- | --- | --- | --- | --- | --- | --- | --- |
| Indirect effect | Metabolic Factor → Susceptibility Values → Cognitive Function | -0.048 | 0.022 | -2.187 | 0.029 | -0.096 | -0.008 | -0.051 |
| Total effect | Metabolic Factor → Cognitive Function | -0.037 | 0.054 | -0.673 | 0.501 | -0.148 | 0.077 | -0.038 |

Est (Estimate): The estimated value of the effect, indicating the magnitude and direction of the relationship. Se (Standard Error): The standard error of the estimated effect, measuring the precision of the estimate. Z: The Z-value, calculated as the estimate divided by the standard error, used to assess the statistical significance of the effect. P-value: The p-value for testing the significance of the effect. CI.lower / CI.upper: The lower and upper bounds of the confidence interval for the effect. Std.Est (Standardized Estimate): The standardized version of the effect size.

**Table S6** : Sensitivity analysis model including blood lipid markers

| Lhs | Rhs | Est | SE | Z | P-value | CI.lower | CI.upper | Std. (All) |
| --- | --- | --- | --- | --- | --- | --- | --- | --- |
| Susceptibility Values | pCAU rh | 1.000^*^ | 0.000 |  |  | 1.000 | 1.000 | 0.792 |
| Susceptibility Values | aPUT rh | 1.172 | 0.091 | 12.928 | <.001 | 1.010 | 1.383 | 0.930 |
| Susceptibility Values | pPUT rh | 1.172 | 0.095 | 12.377 | <.001 | 1.003 | 1.379 | 0.930 |
| Cognitive Function | SCWT | 1.000^*^ | 0.000 |  |  | 1.000 | 1.000 | 0.561 |
| Cognitive Function | TMT | 1.374 | 0.178 | 7.712 | <.001 | 1.088 | 1.812 | 0.775 |
| Cognitive Function | SDMT | 1.677 | 0.277 | 6.049 | <.001 | 1.238 | 2.372 | 0.951 |
| Metabolic Factor | FBG | 1.000^*^ | 0.000 |  |  | 1.000 | 1.000 | 0.642 |
| Metabolic Factor | HbA1c | 1.683 | 0.540 | 3.115 | 0.002 | 1.105 | 3.104 | 1.081 |
| Metabolic Factor | HbA1c squared | 2.180 | 0.536 | 4.066 | <.001 | 1.225 | 3.355 | 0.699 |
| Metabolic Factor | TG | 0.285 | 0.116 | 2.456 | 0.014 | 0.061 | 0.523 | 0.183 |
| Metabolic Factor | HDL | -0.318 | 0.109 | -2.922 | 0.003 | -0.549 | -0.119 | -0.204 |
| Susceptibility Values | Metabolic Factor | 0.215 | 0.086 | 2.484 | 0.013 | 0.062 | 0.397 | 0.175 |
| Susceptibility Values | Age | 0.113 | 0.056 | 2.033 | 0.042 | 0.009 | 0.227 | 0.143 |
| Cognitive Function | Susceptibility Values | -0.249 | 0.055 | -4.515 | <.001 | -0.381 | -0.163 | -0.353 |
| Cognitive Function | Metabolic Factor | -0.079 | 0.061 | -1.298 | 0.194 | -0.212 | 0.026 | -0.091 |
| Cognitive Function | Age | -0.354 | 0.057 | -6.263 | <.001 | -0.471 | -0.241 | -0.634 |

**Abbreviation:** aPUT: Anterior putamen, pCAU: Posterior caudate, rh: Right hemisphere, lh: Left hemisphere, Lhs: Left-Hand Side, Rhs:Right-Hand Side, Est:Estimate, SE:Standard Error, Std.Est:Standardized Estimate, CI: Confidence Interval.

^*^ FBG, SCWT, and pCAU_rh are fixed at 1.000 for model identification purposes. The standard error is zero because these parameters are fixed.

**Table S7** : Direct and indirect effects of the sensitivity analysis model

| **Effect Type** |  | **Est** | **Se** | **Z** | **P-value** | **CI.lower** | **CI.upper** | **Std.Est** |
| --- | --- | --- | --- | --- | --- | --- | --- | --- |
| Indirect effect | Metabolic Factor → Susceptibility Values → Cognitive Function | -0.054 | 0.024 | -2.250 | 0.024 | -0.108 | -0.015 | -0.062 |
| Total effect | Metabolic Factor → Cognitive Function | -0.133 | 0.063 | -2.124 | 0.034 | -0.276 | -0.031 | -0.153 |

Est (Estimate): The estimated value of the effect, indicating the magnitude and direction of the relationship. Se (Standard Error): The standard error of the estimated effect, measuring the precision of the estimate. Z: The Z-value, calculated as the estimate divided by the standard error, used to assess the statistical significance of the effect. P-value: The p-value for testing the significance of the effect. CI.lower / CI.upper: The lower and upper bounds of the confidence interval for the effect. Std.Est (Standardized Estimate): The standardized version of the effect size.

**Table S8** :Comparison of fit indices across models

| **Model** | **χ²** | **Df** | **P-value** | **CFI** | **TLI** | **RMSEA** | **SRMR** | **AIC** | **BIC** |
| --- | --- | --- | --- | --- | --- | --- | --- | --- | --- |
| Original | 83.695 | 31.000 | <.001 | 0.959 | 0.940 | 0.068 | 0.069 | 4673.013 | 4750.856 |
| Alternative | 76.686 | 30.000 | <.001 | 0.963 | 0.945 | 0.072 | 0.070 | 4668.003 | 4749.231 |
| Sensitivity | 206.339 | 50.000 | <.001 | 0.888 | 0.852 | 0.120 | 0.098 | 5895.541 | 5986.922 |

**Table S9** :Linear Regression Models of TMT Performance with Age Interaction Terms for HbA1c and FBG

| Variables | Univariate | | | | |  | Multivariate | | | | |
| --- | --- | --- | --- | --- | --- | --- | --- | --- | --- | --- | --- |
|  | β | S.E | t | *P* | β (95%CI) |  | β | S.E | t | *P* | β (95%CI) |
| Age | 8.06 | 0.99 | 8.10 | **<.001** | 8.06 (6.11 ~ 10.01) |  | 6.66 | 1.15 | 5.78 | **<.001** | 6.66 (4.40 ~ 8.92) |
| HbA1c | 12.26 | 4.73 | 2.59 | **0.010** | 12.26 (2.99 ~ 21.53) |  |  |  |  |  |  |
| FBG | -0.24 | 3.60 | -0.07 | 0.946 | -0.24 (-7.30 ~ 6.81) |  |  |  |  |  |  |
| Age*FBG | 0.14 | 0.06 | 2.40 | **0.017** | 0.14 (0.03 ~ 0.25) |  | -0.15 | 0.07 | -2.03 | **0.043** | -0.15 (-0.29 ~ -0.01) |
| Age*HbA1c | 0.36 | 0.06 | 5.65 | **<.001** | 0.36 (0.24 ~ 0.49) |  | 0.24 | 0.10 | 2.56 | **0.011** | 0.24 (0.06 ~ 0.43) |
| BMI | -0.27 | 2.79 | -0.10 | 0.924 | -0.27 (-5.73 ~ 5.20) |  |  |  |  |  |  |
| Education | -1.53 | 2.14 | -0.72 | 0.474 | -1.53 (-5.72 ~ 2.66) |  |  |  |  |  |  |
| aPUT rh | 0.78 | 0.33 | 2.34 | **0.020** | 0.78 (0.13 ~ 1.44) |  |  |  |  |  |  |
| pPUT rh | 0.76 | 0.30 | 2.55 | **0.011** | 0.76 (0.18 ~ 1.35) |  |  |  |  |  |  |
| pCAU rh | 0.93 | 0.32 | 2.95 | **0.004** | 0.93 (0.31 ~ 1.55) |  | 0.70 | 0.29 | 2.42 | **0.016** | 0.70 (0.13 ~ 1.26) |
| Gender |  |  |  |  |  |  |  |  |  |  |  |
| Male |  |  |  |  | 0.00 (Reference) |  |  |  |  |  |  |
| Female | 19.93 | 17.56 | 1.13 | 0.258 | 19.93 (-14.49 ~ 54.34) |  |  |  |  |  |  |
| T2DM |  |  |  |  |  |  |  |  |  |  |  |
| No |  |  |  |  | 0.00 (Reference) |  |  |  |  |  |  |
| Yes | 17.07 | 17.57 | 0.97 | 0.332 | 17.07 (-17.37 ~ 51.52) |  |  |  |  |  |  |
| Smoke |  |  |  |  |  |  |  |  |  |  |  |
| No |  |  |  |  | 0.00 (Reference) |  |  |  |  |  |  |
| Yes | 11.75 | 20.32 | 0.58 | 0.564 | 11.75 (-28.08 ~ 51.58) |  |  |  |  |  |  |
| Drink |  |  |  |  |  |  |  |  |  |  |  |
| No |  |  |  |  | 0.00 (Reference) |  |  |  |  |  |  |
| Yes | 5.69 | 18.66 | 0.30 | 0.761 | 5.69 (-30.88 ~ 42.25) |  |  |  |  |  |  |
| Hypertension |  |  |  |  |  |  |  |  |  |  |  |
| No |  |  |  |  | 0.00 (Reference) |  |  |  |  |  |  |
| Yes | 43.27 | 17.61 | 2.46 | **0.015** | 43.27 (8.77 ~ 77.78) |  |  |  |  |  |  |
| CI: Confidence Interval | | | | | | | | | | | |

**Table S10** :Linear Regression Models of SCWT Performance with Age Interaction Terms for HbA1c and FBG

| Variables | Univariate | | | | |  | Multivariate | | | | |
| --- | --- | --- | --- | --- | --- | --- | --- | --- | --- | --- | --- |
|  | β | S.E | t | *P* | β (95%CI) |  | β | S.E | t | *P* | β (95%CI) |
| Age | 2.18 | 0.40 | 5.45 | **<.001** | 2.18 (1.39 ~ 2.96) |  |  |  |  |  |  |
| HbA1c | 7.42 | 1.74 | 4.28 | **<.001** | 7.42 (4.02 ~ 10.82) |  |  |  |  |  |  |
| FBG | 3.16 | 1.34 | 2.37 | **0.019** | 3.16 (0.54 ~ 5.79) |  | -12.41 | 3.55 | -3.50 | **<.001** | -12.41 (-19.36 ~ -5.45) |
| Age*FBG | 0.09 | 0.02 | 4.46 | **<.001** | 0.09 (0.05 ~ 0.13) |  | 0.18 | 0.07 | 2.71 | **0.007** | 0.18 (0.05 ~ 0.32) |
| Age*HbA1c | 0.15 | 0.02 | 6.52 | **<.001** | 0.15 (0.11 ~ 0.20) |  | 0.11 | 0.04 | 3.09 | **0.002** | 0.11 (0.04 ~ 0.18) |
| BMI | 0.56 | 1.05 | 0.53 | 0.595 | 0.56 (-1.50 ~ 2.61) |  |  |  |  |  |  |
| Education | -1.19 | 0.80 | -1.49 | 0.138 | -1.19 (-2.76 ~ 0.38) |  |  |  |  |  |  |
| aPUT rh | 0.36 | 0.12 | 2.84 | **0.005** | 0.36 (0.11 ~ 0.60) |  |  |  |  |  |  |
| pPUT rh | 0.33 | 0.11 | 2.96 | **0.003** | 0.33 (0.11 ~ 0.55) |  |  |  |  |  |  |
| pCAU rh | 0.46 | 0.12 | 3.90 | **<.001** | 0.46 (0.23 ~ 0.69) |  | 0.31 | 0.11 | 2.78 | **0.006** | 0.31 (0.09 ~ 0.52) |
| Gender |  |  |  |  |  |  |  |  |  |  |  |
| Male |  |  |  |  | 0.00 (Reference) |  |  |  |  |  |  |
| Female | -16.67 | 6.53 | -2.55 | **0.011** | -16.67 (-29.47 ~ -3.87) |  |  |  |  |  |  |
| T2DM |  |  |  |  |  |  |  |  |  |  |  |
| No |  |  |  |  | 0.00 (Reference) |  |  |  |  |  |  |
| Yes | 18.51 | 6.51 | 2.84 | **0.005** | 18.51 (5.75 ~ 31.26) |  |  |  |  |  |  |
| Smoke |  |  |  |  |  |  |  |  |  |  |  |
| No |  |  |  |  | 0.00 (Reference) |  |  |  |  |  |  |
| Yes | 20.67 | 7.52 | 2.75 | **0.006** | 20.67 (5.93 ~ 35.42) |  |  |  |  |  |  |
| Drink |  |  |  |  |  |  |  |  |  |  |  |
| No |  |  |  |  | 0.00 (Reference) |  |  |  |  |  | 0.00 (Reference) |
| Yes | 18.30 | 6.91 | 2.65 | **0.009** | 18.30 (4.75 ~ 31.85) |  | 16.21 | 6.41 | 2.53 | **0.012** | 16.21 (3.66 ~ 28.77) |
| Hypertension |  |  |  |  |  |  |  |  |  |  |  |
| No |  |  |  |  | 0.00 (Reference) |  |  |  |  |  |  |
| Yes | 6.90 | 6.70 | 1.03 | 0.304 | 6.90 (-6.22 ~ 20.03) |  |  |  |  |  |  |
| CI: Confidence Interval | | | | | | | | | | | |

**Table S11** :Linear Regression Models of SDMT Performance with Age Interaction Terms for HbA1c and FBG

| Variables | Univariate | | | | |  | Multivariate | | | | |
| --- | --- | --- | --- | --- | --- | --- | --- | --- | --- | --- | --- |
|  | β | S.E | t | *P* | β (95%CI) |  | β | S.E | t | *P* | β (95%CI) |
| Age | -1.21 | 0.09 | -13.83 | **<.001** | -1.21 (-1.39 ~ -1.04) |  |  |  |  |  |  |
| HbA1c | -1.58 | 0.50 | -3.19 | **0.002** | -1.58 (-2.55 ~ -0.61) |  | 8.82 | 0.82 | 10.71 | **<.001** | 8.82 (7.20 ~ 10.43) |
| FBG | -0.25 | 0.38 | -0.67 | 0.506 | -0.25 (-1.00 ~ 0.49) |  |  |  |  |  |  |
| Age*FBG | -0.03 | 0.01 | -4.41 | **<.001** | -0.03 (-0.04 ~ -0.01) |  |  |  |  |  |  |
| Age*HbA1c | -0.05 | 0.01 | -8.15 | **<.001** | -0.05 (-0.06 ~ -0.04) |  | -0.16 | 0.01 | -13.72 | **<.001** | -0.16 (-0.18 ~ -0.14) |
| BMI | -0.43 | 0.29 | -1.48 | 0.141 | -0.43 (-1.01 ~ 0.14) |  |  |  |  |  |  |
| Education | 0.18 | 0.23 | 0.81 | 0.419 | 0.18 (-0.26 ~ 0.63) |  |  |  |  |  |  |
| aPUT rh | -0.10 | 0.03 | -2.86 | **0.005** | -0.10 (-0.17 ~ -0.03) |  |  |  |  |  |  |
| pPUT rh | -0.10 | 0.03 | -3.10 | **0.002** | -0.10 (-0.16 ~ -0.04) |  |  |  |  |  |  |
| pCAU rh | -0.11 | 0.03 | -3.25 | **0.001** | -0.11 (-0.17 ~ -0.04) |  | -0.06 | 0.02 | -2.54 | **0.012** | -0.06 (-0.11 ~ -0.01) |
| Gender |  |  |  |  |  |  |  |  |  |  |  |
| Male |  |  |  |  | 0.00 (Reference) |  |  |  |  |  |  |
| Female | 2.21 | 1.86 | 1.19 | 0.236 | 2.21 (-1.43 ~ 5.84) |  |  |  |  |  |  |
| T2DM |  |  |  |  |  |  |  |  |  |  |  |
| No |  |  |  |  | 0.00 (Reference) |  |  |  |  |  |  |
| Yes | -4.14 | 1.84 | -2.25 | **0.025** | -4.14 (-7.75 ~ -0.54) |  |  |  |  |  |  |
| Smoke |  |  |  |  |  |  |  |  |  |  |  |
| No |  |  |  |  | 0.00 (Reference) |  |  |  |  |  |  |
| Yes | -3.97 | 2.13 | -1.86 | 0.064 | -3.97 (-8.14 ~ 0.20) |  |  |  |  |  |  |
| Drink |  |  |  |  |  |  |  |  |  |  |  |
| No |  |  |  |  | 0.00 (Reference) |  |  |  |  |  |  |
| Yes | -3.02 | 1.96 | -1.54 | 0.125 | -3.02 (-6.86 ~ 0.82) |  |  |  |  |  |  |
| Hypertension |  |  |  |  |  |  |  |  |  |  |  |
| No |  |  |  |  | 0.00 (Reference) |  |  |  |  |  |  |
| Yes | -6.24 | 1.84 | -3.39 | **<.001** | -6.24 (-9.84 ~ -2.63) |  |  |  |  |  |  |
| CI: Confidence Interval | | | | | | | | | | | |

**Table S12** : QSM values for all 32 subcortical ROIs in both T2DM and HC groups

| Subcortical ROIs | HC | T2DM | Statistic | P.value |
| --- | --- | --- | --- | --- |
| aHIP_rh | 5.58 (-1.64, 14.24) | 5.71 (-1.20, 14.41) | χ² = 0.81 | 0.371 |
| pHIP_rh | -9.87 (-17.10, -2.65) | -6.10 (-14.73, -0.47) | χ² = 3.06 | 0.082 |
| lAMY_rh | -5.91 (-15.64, 1.67) | -3.27 (-15.07, 2.86) | χ² = 1.01 | 0.317 |
| mAMY_rh | -7.75 (-21.15, 3.09) | -7.21 (-15.88, 2.47) | χ² = 2.10 | 0.149 |
| THA_DP_rh | 18.12 ± 16.18 | 22.29 ± 16.61 | χ² = 3.58 | 0.060 |
| THA_VP_rh | -7.02 ± 12.16 | -3.71 ± 10.69 | χ² = 4.62 | 0.033 |
| THA_VA_rh | -7.25 (-18.07, -0.04) | -1.34 (-9.64, 3.68) | χ² = 6.99 | 0.009 |
| THA_DA_rh | -4.99 ± 12.39 | 0.18 ± 13.46 | χ² = 8.85 | 0.003 |
| NAc_shell_rh | 26.14 (9.60, 46.98) | 41.01 (15.30, 59.49) | χ² = 6.53 | 0.011 |
| NAc_core_rh | 36.47 ± 26.07 | 42.68 ± 26.48 | χ² = 3.09 | 0.080 |
| pGP_rh | 101.73 (77.18, 121.09) | 101.54 (68.11, 125.81) | χ² = 0.01 | 0.939 |
| aGP_rh | 160.77 (122.14, 192.88) | 166.27 (115.30, 204.59) | χ² = 0.04 | 0.851 |
| aPUT_rh | 40.76 ± 26.10 | 51.98 ± 25.58 | χ² = 10.41 | 0.001 |
| pPUT_rh | 43.58 (24.34, 61.84) | 54.52 (37.19, 72.82) | χ² = 8.11 | 0.005 |
| aCAU_rh | 57.76 (40.35, 78.41) | 70.99 (43.90, 85.15) | χ² = 2.42 | 0.121 |
| pCAU_rh | 39.01 ± 25.16 | 51.84 ± 28.72 | χ² = 12.48 | < .001 |
| aHIP_lh | 5.51 (-1.54, 15.22) | 5.16 (-0.35, 13.06) | χ² = 0.04 | 0.846 |
| pHIP_lh | -8.99 (-15.93, -1.87) | -6.98 (-12.86, -0.34) | χ² = 2.18 | 0.141 |
| lAMY_lh | -3.97 (-14.24, 8.97) | -1.69 (-15.80, 5.22) | χ² = 0.02 | 0.878 |
| mAMY_lh | -8.67 (-20.10, 2.78) | -8.21 (-18.52, 4.27) | χ² = 0.24 | 0.624 |
| THA_DP_lh | 14.38 (5.32, 26.93) | 17.63 (6.29, 31.94) | χ² = 1.54 | 0.216 |
| THA_VP_lh | -8.25 (-14.76, -1.69) | -5.93 (-13.16, -0.73) | χ² = 1.36 | 0.245 |
| THA_VA_lh | -5.07 (-17.40, 2.48) | -3.46 (-10.35, 3.57) | χ² = 3.31 | 0.070 |
| THA_DA_lh | -5.29 ± 12.38 | -2.05 ± 12.81 | χ² = 3.65 | 0.057 |
| NAc_shell_lh | 20.38 (7.25, 39.18) | 28.59 (16.78, 48.49) | χ² = 3.66 | 0.057 |
| NAc_core_lh | 32.59 ± 24.72 | 34.84 ± 23.63 | χ² = 0.48 | 0.488 |
| pGP_lh | 110.59 (82.09, 140.44) | 125.14 (100.21, 148.27) | χ² = 2.16 | 0.143 |
| aGP_lh | 162.40 (125.36, 208.30) | 179.93 (125.43, 221.38) | χ² = 0.32 | 0.574 |
| aPUT_lh | 37.15 ± 26.10 | 44.16 ± 26.49 | χ² = 3.92 | 0.049 |
| pPUT_lh | 42.27 (25.61, 62.96) | 51.01 (29.27, 73.76) | χ² = 2.45 | 0.119 |
| aCAU_lh | 58.52 (39.42, 72.61) | 62.30 (42.23, 77.24) | χ² = 0.70 | 0.404 |
| pCAU_lh | 37.48 ± 21.87 | 45.07 ± 27.14 | χ² = 5.25 | 0.023 |

QSM values (ppb)

Variables were first assessed for normality using the Shapiro–Wilk test (p > 0.05 indicating approximate normality).

Variables meeting the normality criterion (Shapiro–Wilk p > 0.05) are presented as mean ± standard deviation (SD).

Variables violating normality (Shapiro–Wilk p ≤ 0.05) are presented as median (interquartile range [IQR]).

Group differences for continuous variables were evaluated using Kruskal-Wallis test. A p-value < 0.05 was considered statistically significant.

**Figure S1: Non-linear relationship curve between susceptibility values of subcortical nuclei and FBG**


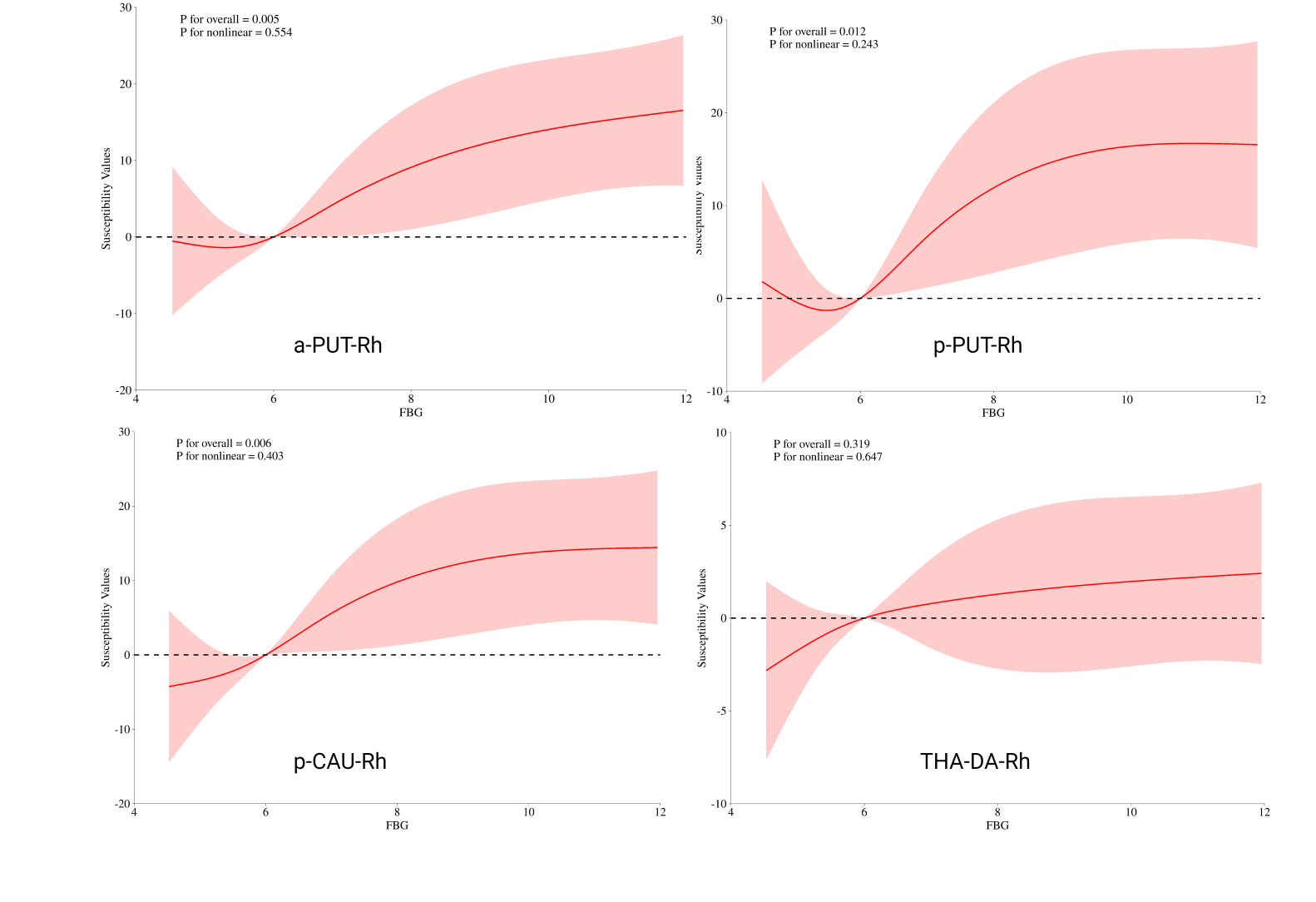


aPUT: Anterior putamen, pCAU: Posterior caudate, THA-DA: Dorsoanterior thalamus, rh: Right hemisphere, lh: Left hemisphere
